# The Impact of Antibiotic Prophylaxis on Antibiotic Resistance, Clinical Outcomes, and Costs in Adult Hemato-Oncological and Surgical Patients: A Systematic Review and Meta-Analysis

**DOI:** 10.3390/antibiotics14090853

**Published:** 2025-08-22

**Authors:** Marissa Rink, Beryl Primrose Gladstone, Lea Ann Nikolai, Michael Bitzer, Evelina Tacconelli, Siri Göpel

**Affiliations:** 1 Department of Internal Medicine I, University Hospital Tübingen, 72076 Tübingen, Germany; marissa.rink@med.uni-tuebingen.de (M.R.); primrose.beryl@med.uni-tuebingen.de (B.P.G.); lea.nikolai@med.uni-tuebingen.de (L.A.N.); m.bitzer@med.uni-tuebingen.de (M.B.); 2 DZIF-Clinical Research Unit, German Centre for Infection Research, 38108 Braunschweig, Germany; 3 Department of Diagnostics and Public Health, Section of Infectious Diseases, University of Verona, 37124 Verona, Italy; evelina.tacconelli@univr.it

**Keywords:** antibiotic resistance, antibiotic prophylaxis, systematic review, secondary burden, meta-analysis

## Abstract

Background/Objectives: While antibiotic prophylaxis is crucial for preventing infections, its impact on the development of antibiotic-resistant infections and clinical outcomes remains underexplored. We aimed to systematically assess the impact of medical and surgical antibiotic prophylaxis (SAP) on the development of antibiotic-resistant infections, clinical outcomes, and costs. Methods: A systematic review and meta-analysis of the effect of antibiotic prophylaxis on antibiotic-resistant infections, mortality, length of hospital stay, and/or costs was conducted in hemato-oncological or surgical patient populations. Pooled estimates of the relative risk (RR) or weighted mean difference (WMD) were derived using random-effect meta-analysis. Results: Of 10,409 screened studies, 109 (30%) comprising 131,519 patients were included. In 55 hemato-oncological studies, prophylaxis significantly reduced Gram-negative infections (RR: 0.51; 95% CI: 0.45 to 0.59) without an effect on mortality (RR = 1.01; 95% CI: 0.89 to 1.15), while the risk of developing an infection resistant to prophylactic antibiotics during hospitalization was doubled (RR: 2.05; 95% CI: 1.88 to 2.23). The length of hospitalization was reduced by 1.85 days. Among 54 surgical studies, SAP lowered surgical-site infections (RR: 0.58; 95% CI: 0.49 to 0.69). Extending prophylaxis beyond the recommended duration did not improve infection rates (RR: 1.10; 95% CI: 0.98 to 1.24). No association was demonstrated between prophylaxis adjusted by colonization status and the development of resistant infections. Conclusion: Though proven beneficial, our results highlight the critical need for targeted antibiotic stewardship programs (ASPs) in both settings. A meticulous risk assessment balancing the benefits of preventing life-threatening infections against the risk of driving antimicrobial resistance, and a tailored ASP, is urgently needed for hemato-oncological patients.

## 1. Introduction

The global burden of antimicrobial resistance (AMR) is undeniably substantial [1]. Global estimates suggest that 4.95 million deaths were associated with AMR in 2019 [2], with 1.27 million of these directly attributable to bacterial AMR [3]. A primary driver of AMR is the inappropriate or inadequate usage of antibiotics, whether for therapeutic or prophylactic purposes.

Prophylactic antibiotic use accounts for 24.9% of antimicrobial consumption in European acute care hospitals [4]. Surgical antibiotic prophylaxis (SAP) is employed to prevent surgical site infections (SSIs), which are among the most common hospital-acquired infections. SSIs are associated with increased mortality and treatment costs, and prolonged hospital stays [5]. The benefit of perioperative antibiotic prophylaxis was first demonstrated by data generated in the 1960s [6]. Since then, guidelines and recommendations for clinical practice have been established, tailored to patient setting, type of surgery, and target bacteria.

The choice of antibiotic agent depends on the expected pathogen spectrum. SSIs are most commonly caused by Gram-positive skin flora, which can be effectively prevented with first-generation cephalosporins. Depending on the type of surgery and anatomic location, additional coverage of Gram-negative bacteria or Anaerobes might also be necessary [7]. However, the appropriateness of antibiotic use extends beyond the choice of antibiotic to encompass its timing, duration, and dosage. As early as 1979, it was found that the optimal timing of antibiotic therapy perioperatively is crucial and that prolonged SAP postoperatively does not improve infection rates [8]. Nevertheless, a high prevalence of extended postoperative antibiotic usage is still commonly reported globally [9,10,11], and heterogeneous adherence to guidelines [12,13,14] remains a primary target for antimicrobial stewardship interventions [15].

While SAP is typically confined to the perioperative period, mostly as a single intravenous dose administered in a defined interval prior to surgery, antibiotic prophylaxis in the hemato-oncological setting is given orally over a longer duration and often as ambulatory use. The common prophylactic agents are fluoroquinolones to prevent infections predominantly caused by Gram-negative pathogens during the neutropenic period in high-risk hemato-oncological patients, such as patients with acute leukemia or myelodysplastic syndromes, patients in advanced cancer stages, or patients undergoing myeloablative regimens [16]. Although prophylaxis in hemato-oncology aims to prevent severe infections in immunocompromised patients, its potential impact on the development of antibiotic resistance is still undefined.

Despite the well-established antibiotic prophylaxis—especially SAP—for several decades, increasing rates of AMR and its consequences have endangered its efficacy. This is evident in a meta-analysis of randomized controlled trials [17], which showed the declining efficacy of antibiotic prophylaxis in colorectal surgery patients. Several systematic reviews have investigated specific prophylactic antibiotics, evaluating their efficacy in preventing various clinical outcomes and AMR development [18,19,20,21,22], resulting in a wide spectrum of evidence ranging from low to high risk.

We conducted a systematic review and meta-analysis to investigate the impact of prophylactic antibiotics on the incidence of infections as well as the development of infections due to antibiotic-resistant bacteria among adult hemato-oncological and surgical patients. The secondary objectives were to assess the effect of prophylaxis on mortality, length of stay, and healthcare costs.

## 2. Methods

A systematic review targeting antibiotic prophylaxis in the hemato-oncological setting and surgical prophylaxis was conducted and reported in accordance with the *Cochrane Handbook of Systematic Reviews* and the PRISMA statement (Supplementary Material).

### 2.1. Literature Search Strategy

A literature search was conducted on 24 April 2024 in PubMed, Web of Science, and the Cochrane Library. The study protocol was registered in PROSPERO–CRD42021267613 (https://www.crd.york.ac.uk/PROSPERO/view/CRD42021267613 (accessed on 31 March 2025)) [23]. The search terms combined antibiotic prophylaxis, antibiotic resistance, and at least one of the predefined outcomes (mortality, length of stay, or cost) with specific search terms for the settings of hemato-oncology or surgery (Supplementary Text S1).

### 2.2. Inclusion and Exclusion Criteria

We included all clinical studies with at least one control group assessing antibiotic prophylaxis in surgical or hemato-oncological settings. Interventional and observational studies providing data on prophylactic antibiotic usage, antibiotic resistance, and at least one clinical outcome (infection rate and susceptibility pattern of etiological agents, mortality, morbidity, and length of hospital stay) and/or healthcare costs were considered eligible. Studies conducted exclusively in a pediatric setting or those focusing only on colonization of antibiotic-resistant pathogens were excluded. Surgical studies on patients undergoing solid organ transplantation were excluded due to the often-intensive antibiotic pretreatment in this population. To ensure homogeneity given changes in guidelines, susceptibility testing methods, surgical techniques, and chemotherapeutic agents over time, only studies published after 1990 were included.

### 2.3. Data Extraction

The eligibility of trials was independently assessed by two different reviewers. Discrepancies were sorted, and the reason for exclusion was noted. Researchers were not blinded to the study authors or location. The data related to the publication, study characteristics, patient characteristics, prophylactic regimen, control group, clinical outcomes, and costs were extracted into a structured database within REDCap [24]. Infection types, antibiotic resistance, and all-cause and infection-related mortality, along with their defined time-periods, were recorded as reported by the original investigators. Prophylactic regimens were classified according to antibiotic classes.

### 2.4. Data Analysis

The data analysis and presentation of results were performed separately for hemato-oncological and surgical studies. The primary outcome was the relative risk (RR) of acquiring resistant bacterial infections for patients receiving antibiotic prophylaxis compared with those not receiving any prophylaxis or a prophylaxis differing in antibiotic type, dosage, duration, or mode of administration. Secondary outcomes included the relative risk of SSI among surgical patients or any infection in hemato-oncological patients; all-cause and/or attributable mortality; and the weighted mean difference (WMD) for length of hospital stay in days among patients receiving prophylaxis compared with those not receiving any prophylaxis.

Overall pooled effect estimates of the impact of prophylaxis were calculated for hemato-oncological and surgical patients individually using random-effect meta-analysis and expressed as RR or WMD with a 95% CI, in the presence of at least 3 studies providing the outcome measure. These were visualized in the form of forest plots and summary forest plots. The duplication of study estimates was avoided by using the most representative outcome in each study (e.g., if a study reported an overall resistance rate and then specified it for different antibiotic classes or bacteria, the overall rate was preferred). Outcome measures based on fewer than 10 patients/infections/samples/isolates were excluded from the analysis.

Heterogeneity was studied among the following subgroups/study characteristics: prophylactic agent, isolated bacteria, infection type, country, publication year, and another comparison group, where applicable. Additional subgroups of interest were the underlying hemato-oncological disease and chemotherapy/stem cell transplantation, type of surgery, and short versus extended prophylaxis. The definition of extended prophylaxis was based on the most recent guidelines on that specific surgery, as referred to by the study authors. Subgroup analysis was performed wherever there were more than two studies available in a group. Heterogeneity was evaluated by using I^2^ statistics and meta-regression. Statistical analyses were performed using Stata/SE 15 [25].

Risk of bias was assessed using the Joanna Briggs Institute (JBI)’s critical appraisal tools [26].

## 3. Results

### 3.1. Study Selection and Study Characteristics

**Figure 1:**
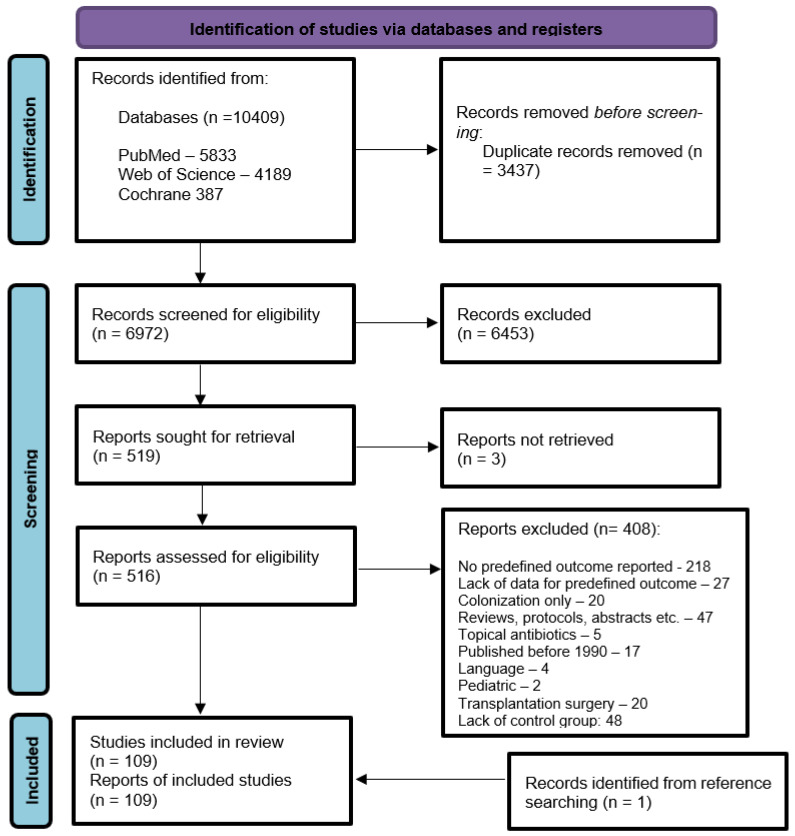
PRISMA flow chart diagram for literature search and study inclusion and exclusion.

Our search identified 10,409 studies, of which 516 (5.0%) were found eligible after title–abstract screening. Upon full-text screening, 109 (2.1%) studies [27,28,29,30,31,32,33,34,35,36,37,38,39,40,41,42,43,44,45,46,47,48,49,50,51,52,53,54,55,56,57,58,59,60,61,62,63,64,65,66,67,68,69,70,71,72,73,74,75,76,77,78,79,80,81,82,83,84,85,86,87,88,89,90,91,92,93,94,95,96,97,98,99,100,101,102,103,104,105,106,107,108,109,110,111,112,113,114,115,116,117,118,119,120,121,122,123,124,125,126,127,128,129,130,131,132,133,134,135] involving 131,519 patients were included in the qualitative and quantitative analysis (Figure 1 and Supplementary Table S1). In total, 55 (50.5) hemato-oncological and 54 (49.5%) surgical studies were included. The majority of studies originated from the USA (28 (26%)). Over half were retrospective cohort studies (51.4%), and the majority were monocentric (92 (84.4%)). The average age of the patients ranged from 35 to 72 years (n = 92), and most hemato-oncological studies (38/45 (84%)) defined neutropenia as an absolute neutrophil count (ANC) below 500 cells/µL, and 6 (13%) defined it as below 1000 cells/µL. The characteristics of the included studies are provided in Table 1.

The comparison groups varied, including patients not receiving prophylaxis (48 (45.0%)), patients receiving a different antibiotic regimen (44 (40.4%)), and patients with different colonization status (7 (5.5%)); 10 studies (9.2%) had more than one comparison group. A total of 3 studies (43%) used targeted prophylaxis based on the screening results of patients’ colonization status. Comparison groups including patients receiving no prophylaxis were mainly found in the hemato-oncological setting (44 (80.0%) vs. 6 (11.0%); *p* < 0.001), while in the surgical setting, comparison between different prophylactic regimens was predominant (48 (87.3%) versus 12 (22.2%)). The short SAP regimens compared against extended SAP included perioperative (n = 10), 24 h postoperative (n = 3), or <48 h postoperative (n = 1), as recommended by guidelines. Frequently studied antibiotic classes were cephalosporins (27 (50.0%)) in the surgical setting and quinolones (51 (92.7%)) in the hemato-oncological setting, either alone or in combination with another antibiotic (for details, see Supplementary Section S2). In total, 438 outcomes were reported. The most frequently reported clinical outcome in both settings was the overall rate of infection (including AMR), followed by mortality. Cost was investigated in 4 (3.6%) studies. For surgical studies, outcome assessment timeframes pertained to the duration of hospital stay in 136 (78.2%) outcomes; 30-day outcomes in 59 (33.9%); and long-term assessment (≥90 days) in 33 (19%). In hemato-oncological studies, 92.3% of the outcomes were assessed during hospital stay, with 30-day outcomes in 13 (5%) and long-term assessment (≥90 days) in 20 (7.7%).

### 3.2. Impact of Antibiotic Prophylaxis Among Hemato-Oncological Patients

#### 3.2.1. Risk of Infection

The incidence risk of infection was reported in 37 studies, providing 86 outcomes from 16,539 patients. An overview of the results is given in Table 2. A significantly lower risk of infection due to Gram-negative bacteria was observed with prophylaxis (RR = 0.51; 95% CI: 0.45 to 0.59; *p* < 0.001; I^2^ = 62%; p_het_ < 0.001). Conversely, the risk of infections due to Gram-positive bacteria was not different among patients receiving prophylaxis or not (RR: 1.06; 95% CI: 0.95 to 1.19; *p* = 0.28; I^2^ = 78%; p_het_ < 0.001). Quinolone prophylaxis significantly reduced the overall risk of infection by an estimated RR of 0.86 (95% CI: 0.82 to 0.90; *p* < 0.001; I^2^ = 79%; p_het_ < 0.001).

#### 3.2.2. Antibiotic Resistance Rates Among Infections

**Figure 2:**
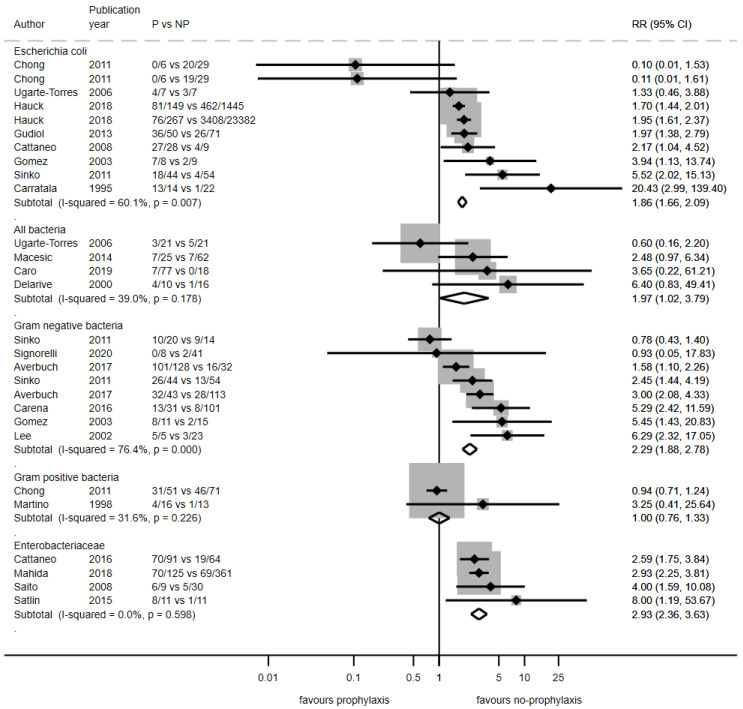
Forest plot of relative risks of resistance against the used prophylactic agent among bacterial infections in hemato-oncological patients receiving prophylaxis compared with those not receiving prophylaxis in studies published between 1991 and 2024 (n = 21). Note: P vs. NP refers to number of resistant bacterial infections/total number of bacterial infections in prophylaxis arm versus no-prophylaxis arm. RR, relative risk; 95% CI, 95% confidence interval.

Assessment of resistance in the etiological agent of infections developed after starting medical prophylaxis was reported in 33 studies, including 34,385 patients and reporting 87 outcomes. The pooled RR to develop an infection resistant to the antibiotic used for prophylaxis during hospitalization was 2.05 (95% CI: 1.88 to 2.23; *p* < 0.0001; I^2^ = 73%; p_het_ < 0.001). The risk was higher for Gram-negative (RR: 2.14; 95% CI: 1.95 to 2.34; *p* < 0.0001; I^2^ = 66%; p_het_ < 0.001) than Gram-positive (RR: 1.13; 95% CI: 0.87 to 1.46; *p* = 0.353; I^2^ = 54%; p_het_ = 0.09) bacteria. Resistance to prophylactic antibiotics in Enterobacterales showed an RR of 2.93 (95% CI: 2.36 to 3.63; *p* < 0.001; I^2^ = 0%; p_het_ = 0.60) and was 1.87 (95% CI: 1.66 to 2.10; *p* < 0.001; I^2^ = 56%; p_het_ = 0.01) in *Escherichia coli*, as shown in Figure 2. Among patients receiving quinolone prophylaxis, the RR for infection with a quinolone-resistant pathogen was 2.04 (95% CI: 1.87 to 2.22; *p* < 0.001; I^2^ = 73%; p_het_ < 0.001) compared with patients not receiving any prophylaxis. Geographical variations as well as time trends were observed (Supplementary Figures S1 and S2). An overview of the results is given in Table 3.

Data on general resistance to any antibiotic could be studied in seven studies, all with fluoroquinolone prophylaxis: three studies reported rates of ESBL-producing bacteria resulting in an RR of 1.82 (95% CI: 1.04 to 3.18; *p* = 0.037; I^2^ = 0%; p_het_ = 0.44), and four studies reported an RR of 1.74 (95% CI: 1.32 to 2.30; *p* <0.001; I^2^ = 87%; p_het_ < 0.001) for multidrug-resistant bacteria (authors definition, Supplementary Table S2).

#### 3.2.3. Mortality

**Figure 3:**
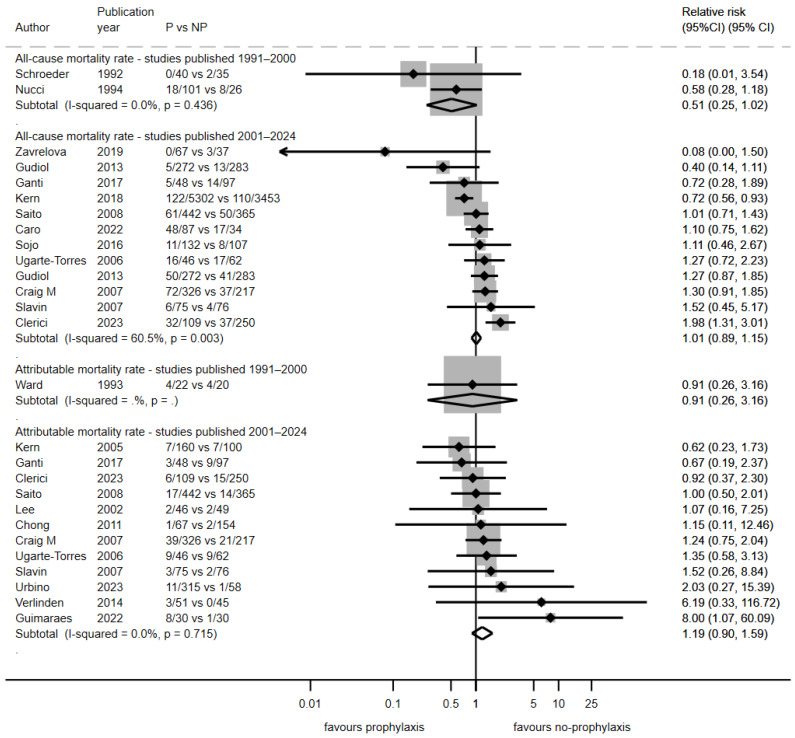
Forest plot of relative risks of mortality in hemato-oncological patients receiving prophylaxis compared with those not receiving prophylaxis reported between 1991 and 2024, classified by type of mortality and publication year (n = 20). Note: P vs. NP refers to number of resistant bacterial infections/total number of bacterial infections in prophylaxis arm versus no-prophylaxis arm. RR, relative risk; 95% CI, 95% confidence interval.

Mortality was analyzed in 22 studies with 30 outcomes involving 13,701 patients. All-cause mortality and attributable mortality during hospitalization were not different between patients receiving prophylaxis compared with those with no prophylaxis (RR = 1.01 (95% CI 0.89 to 1.15; *p* = 0.85; I^2^ = 58%; p_het_ = 0.005) and RR = 1.18 (95% CI: 0.89 to 1.56; *p* = 0.25; I^2^ = 0%; p_het_ = 0.78), respectively). Sensitivity analysis by excluding studies published before 2020 to account for changing antibiotic availability showed similar results (Figure 3).

#### 3.2.4. Length of Hospital Stay

Length of hospital stay (LOS) was reported as an outcome in 8 (7.3%) studies involving 833 patients. The pooled estimate showed that there was a shorter LOS by 1.85 (95% CI: 0.31 to 3.40; *p* = 0.02; I^2^ = 0.0%; p_het_ = 0.79) days among hemato-oncological patients receiving prophylaxis (Supplementary Figure S3).

#### 3.2.5. Clinical Outcomes in Patients Colonized with Antibiotic-Resistant Bacteria Before Prophylaxis

Two hemato-oncological studies compared the infection rates among colonized versus non-colonized patients and confirmed the impact of colonization on infection rates. Satlin et al. [113] compared the impact of fluoroquinolone prophylaxis on the development of fluoroquinolone resistance in colonized patients versus non-colonized patients. Colonized patients had a significantly higher risk of BSI (*p* = 0.005) and Gram-negative BSI (*p* < 0.001), while the 100-day mortality rates were similar (9% vs. 7%; *p* = 0.55). Akhmedov et al. [28] studied the development of resistant Gram-negative infections in three groups of patients: colonized with no prophylaxis, non-colonized with no prophylaxis, and non-colonized with fluoroquinolone prophylaxis. Though BSI rates were similar among the three groups (29.3% vs. 27.5% vs. 28.1%), the proportion of Gram-negative BSI was significantly higher in colonized patients (66.2% vs. 32.8%; *p* = 0.037). Thirty-day mortality rates after BSI showed no significant difference.

#### 3.2.6. Costs

In total, 1 study (1.8%) published in 1992 from Germany estimated a one-fifth reduction in costs, including antibiotic prophylaxis, antibiotic treatment of infection, and hospital stay, for patients receiving fluoroquinolone prophylaxis compared with a placebo [115].

### 3.3. Impact of Antibiotic Prophylaxis Among Surgical Patients

Surgical studies were heterogeneous regarding the comparison group and reported outcomes; hence, few pooled effect estimates, based on at least three studies, were generated.

#### 3.3.1. Risk of Infection

The incidence risk of infection was reported in 46 studies with 69 outcomes involving 75,663 patients. Compared with patients receiving no prophylaxis, surgical patients (neurosurgery (n = 2), spinal surgery (n = 2), and breast cancer surgery (n = 1)) receiving prophylaxis had a lower risk of overall infection (n = 5; RR = 0.65 (95% CI: 0.56 to 0.75; *p* < 0.001; I^2^ = 60%; p_het_ = 0.01)) and SSI specifically (n = 5; RR = 0.58 (95% CI: 0.49 to 0.69; *p* < 0.001; I^2^ = 68%; p_het_ = 0.02)) (Supplementary Figure S4). Four studies among patients undergoing either arthroplasty surgeries [87,108,116] or instrumented spinal fusion [89] compared the prophylactic effect of adding vancomycin to cefazolin, the standard prophylaxis, mainly due to increasing rates of MRSA infections. While three studies found a lower SSI rate, a recent Australian RCT [108] reported no lowering, resulting in a non-significant pooled RR of 0.84 (95% CI: 0.68 to 1.05) (*p* = 0.125; I^2^ = 82%; p_het_ = 0.001). Similarly, we found three studies comparing vancomycin with cefazolin as SAP in three different surgical settings due to high MRSA prevalence [54,93,102]. These studies reported similar rates of SSI or prosthetic joint infections in both arms (RR = 0.93 (95% CI: 0.62 to 1.32; *p* = 0.695; I^2^ = NA; p_het_ = 0.901)). Three studies in urological surgery [30,70,84] published between 2009 and 2016 cited a high background risk of quinolone resistance to study alternative regimens (pivmecillinam–amoxicillin/clavulanic acid combination; third-generation cephalosporin, ciprofloxacin, and gentamicin combination) to fluoroquinolone prophylaxis. The data showed a general lowering of infections with a non-significant lowering of resistant infections, as concluded by the authors, favoring the alternative regimens.

#### 3.3.2. Antibiotic Resistance Rates Among Infections

Infections caused by bacteria resistant to the SAP postoperatively were reported in 10 studies, including 32,289 patients and reporting 16 outcomes; however, there were no more than 2 studies studying similar SAP regimens. Two studies provided data on SSIs caused by prophylactic-resistant bacteria among patients in comparison with a no-prophylaxis arm: a prospective cohort study [29] observed that cefazolin prophylaxis in non-instrumental spinal surgery reduced SSIs (RR = 0.35; 95% CI: 0.21 to 0.58) but was associated with a higher rate of cefazolin-resistant infections (RR = 1.91; 95% CI: 1.01 to 3.63). Similarly, a randomized controlled trial of amoxycillin–clavulanate versus no prophylaxis for breast cancer surgery [123] found a non-significant reduction in SSIs (RR = 0.85 (95% CI: 0.64 to 1.13)) with a non-significant increase in MRSA SSIs (RR = 1.98 (95% CI: 0.49 to 7.96)).

#### 3.3.3. Mortality

Mortality outcomes were compared as 1-year all-cause mortality and in-hospital-attributable mortality among patients on prophylaxis and no prophylaxis in one study each of spinal surgery [29] and craniotomy patients [80], respectively. The mortality rates were not significantly different from the no-prophylaxis patients (with RRs of 0.85 (95% CI: 0.12 to 6.0; *p* = 0.75) and 1.2 (95% CI: 0.29 to 4.9; *p* = 0.89), respectively).

#### 3.3.4. Extended Surgical Prophylaxis and Clinical Outcomes

No significant difference was found in overall infection and SSI rates among short versus extended SAP (n = 14; RR = 1.10 (95% CI: 0.98 to 1.24; *p* = 0.12; I^2^ = 24.0%; p_het_ = 0.16) (Supplementary Figure S5). Two studies comparing extended versus short prophylaxis in cardiac [88] and maxillofacial surgery patients [34] reported either higher or similar resistance rates (RRs of cefazolin-resistant CoNS among CoNS = 1.95 (95% CI: 1.03 to 3.69) and cefazolin-resistant *S. aureus* among all *S. aureus* = 1.26 (95% CI: 0. 30 to 5.24); RR of ampicillin/sulbactam-resistant *Staphylococcus* spp. among all *Staphylococcus* Spp. in SSI samples = 0.83 (95% CI: 0.12 to 5.72)). The pooled estimates from three studies comparing extended versus short prophylaxis showed a significantly longer LOS by 1.36 days (95% CI: 0.74 to 1.98).

#### 3.3.5. Clinical Outcomes in Patients Colonized with Antibiotic-Resistant Bacteria Before Surgical Prophylaxis

Five surgical studies investigated the impact of prophylaxis among colonized patients, with ESBL-producing bacteria being the most frequently assessed colonizing bacteria (3/5 (60%)). One surgical study [51] compared ESBL-colonized with non-colonized patients undergoing elective colorectal surgery on cephalosporin/metronidazole combination prophylaxis. The study concluded that carriage of ESBL-producing bacteria more than doubled the risk of SSI (OR = 2.36; 95% CI: 1.50 to 3.71) and increased the likelihood of infections caused by ESBL-producing bacteria (OR = 4.23; 95% CI: 1.70 to 10.56). Yang et al. studied the concordance of pathogens and their resistance patterns from preoperative flora with those of SSI among high-risk patients in clean-contaminated head and neck reconstructive surgery. The study found a rapid and significant increase in clindamycin resistance (RR = 1.75; 95% CI: 1.40 to 2.18) after clindamycin prophylaxis [133].

The impact of prophylaxis adjusted by colonizing bacteria was studied in three surgical studies. Adjusted prophylaxis using either ertapenem or piperacillin/tazobactam was studied among patients undergoing elective colorectal surgery [104] or pancreatic surgery [49]. Both studies concluded that adjusted prophylaxis significantly reduced the rate of SSIs and ESBL infections. There was no significant difference in 30-day mortality [104]. One study [101] assessed the effect of personalized prophylaxis among patients undergoing transrectal biopsy and did not find a reduction in the risk of post-biopsy sepsis. The details are provided in Table 4.

#### 3.3.6. Costs

A total of 3 studies (5.6%) reported a significant reduction in costs of treatment and hospital stay, comparing various prophylactic regimens: ertapenem prophylaxis was found to be more cost-effective than cefotetan in elective colorectal surgery (with a net difference of -USD 2181); short-course prophylaxis was markedly more economical than extended regimens, with costs (in Indian Rupees) of approximately INR 150 compared with INR 1900, respectively; and the addition of gentamicin to standard fluoroquinolone prophylaxis in urological surgery proved beneficial, involving a cost saving of USD 15,700 per 100 patients for treatment, hospital stay, and surgery costs.

### 3.4. Risk-of-Bias Assessment

Most of the studies were of moderate quality, with a higher risk of bias in the areas of usage of optimal analytical techniques (62/109 (56.9%)), in assessment and adjustment for confounders (65/109 (59.6)). The risk-of-bias assessment is presented in Supplementary Figures S6–S9.

## 4. Discussion

Our systematic review and meta-analysis, based on 109 studies comprising 131,519 patients, confirmed that prophylactic antibiotics are associated with a lower risk of infections in hemato-oncological and surgical populations. On the other hand, it highlighted a higher risk of infections due to bacteria resistant to the prophylactic agent, depending on the setting, bacteria, and prophylactic antibiotic. Precisely speaking, antibiotic prophylaxis among hemato-oncological patients reduced the overall rate of Gram-negative infections by 50% and shortened the length of hospital stay by almost two days. However, a general increase in ESBL-producing and multidrug-resistant bacteria among infecting bacteria was noted, by 82% and 34%, respectively, with no difference in Gram-positive bacterial infections and overall mortality rates. In studies involving patients undergoing a wide range of surgeries, SAP resulted in a 42% reduction in SSIs. Extending prophylaxis beyond the recommended period did not improve outcomes but was associated with increased resistance and longer hospital stays by more than a day. Adjusted prophylaxis for patients colonized with resistant organisms did not impact the selection of resistance.

Our findings align with previous systematic reviews on antibiotic prophylaxis in hemato-oncological patients. Gafter-Gvili et al. [18] analyzed 109 randomized/quasi-randomized controlled trials (1973–2010) in afebrile neutropenic patients, observing a significant reduction in infections and mortality with prophylaxis, alongside a 47% higher risk of antibiotic resistance. The authors concluded that the benefit outweighed the harm. Since then, several systematic reviews have studied the impact of antibiotic prophylaxis among hemato-oncological patients [19,20,22,136], confirming the reduction in infection rates. While Mikulska et al. [20] (including studies published between 2006 and 2014) found discordant results with regard to antibiotic resistance, Egan et al. [22] (studies from 1980 to 2018) estimated a three-fold increase in fluoroquinolone resistance rates among studies of fluoroquinolone prophylaxis. Our finding of a two-fold increase in fluoroquinolone resistance corroborates this finding; the relatively lower estimate, compared with theirs, could be attributed to our exclusion of pediatric studies and inclusion of controlled non-randomized studies.

Notably, the significantly lower risk of mortality in hemato-oncology patients on prophylaxis observed by Gafter-Gvili [18] has not been consistently replicated in subsequent meta-analyses, including ours. However, our subgroup analysis showed that earlier studies (published until 2000) frequently showed an impact on mortality. This shift might be explained by the development of broader-spectrum antibiotics in recent decades, which could help overcome resistance, thereby leading to a negligible effect on overall mortality, unless caused by extensively drug-resistant bacteria. The long-term administration of prophylaxis to reduce the risk of bacterial infections in severely immunocompromised scenarios has a definite influence on resistance development. The relatively higher resistance rates, lack of reduction of mortality rates, and presence of adverse effects, in contrast with Gafter-Gvili [18], have instigated the current state, where antibiotic prophylaxis is under debate for hemato-oncological patients. Several national guidelines now reflect a critical discussion of this subject, selectively recommending prophylaxis only for high-risk patients with longer periods of neutropenia and in their first therapy cycle [137] or an individual risk-based assessment to determine the benefit of antibiotic prophylaxis [16].

We found a higher effect of antibiotic prophylaxis on resistance development in Gram-negative infections than in Gram-positive infections in hemato-oncological settings, which seems to reflect the current clinical practice. Gram-negative bacterial infections are usually targeted by prophylaxis in hemato-oncology, as prophylaxis against Gram-positive bacterial infections has been reported to have more adverse effects with a lack of benefit [138]. A potential selection pressure leading to more Gram-positive infections in the long run is being discussed in this context. Our data could not demonstrate an effect on Gram-positive infections, limited by the fact that the studies usually had a short follow-up period.

On a positive note, we observed a significant reduction in the length of hospital stay among hemato-oncological patients and surgical patients on prophylaxis, an effect measure not estimated in any earlier meta-analysis. The reduced LOS, an indirect indicator of substantially lower healthcare costs, along with the direct assessment of lower costs in some studies, adds a crucial economic dimension to the benefits of prophylaxis in both settings.

In the surgical field, prophylaxis is usually confined to 24 h perioperatively, thus preventing infections, a longer antibiotic treatment, and higher selection pressure in the individual patient. The type and anatomical site of surgery further define the infection risk, ranging from very low in clean procedures to high in contaminated procedures, for example, intra-abdominal procedures. Antibiotic prophylaxis is recommended in procedures with clinically relevant contamination risk. Owing to this, we could only find few studies with a non-prophylactic comparison group, all in low-risk surgical settings. Notably, though an overall reduction in infections could be seen with prophylaxis, a doubling of resistant infections was observed in these studies, indicating that even short-term prophylaxis has an effect on the development of resistance. This emphasizes the need for well-informed decision making for antibiotic prophylaxis, especially in procedures with a very low contamination risk.

Though SAP has been well established with guidelines and recommendations [139,140,141], the recent finding of a 5% annual increase in the risk of SSI following colorectal surgery [17] has raised an alarm. Such findings of a loss of effect of SAP to AMR are aggravated by and attributed to the inappropriate use of the antibiotic in its choice, indication, dose, timing, and duration [142]. Despite the recommendations that SAP should be given as single-shot prophylaxis and only perioperatively [141] with few exceptions and strong evidence against extended regimens [9,10,11,12,13,14,143,144], current prophylactic practices involving longer durations of peri- and postoperative regimens were observed in our review.

We observed a loss of efficacy of standard antibiotic prophylaxis, especially in urological settings with high fluoroquinolone resistance. Several guidelines, including those of the European Association of Urology (EAU) [145] and ESCMID/EUCIC [146], have already been adapted to high fluoroquinolone resistance rates. These no longer recommend fluoroquinolones and highlight the need for continuous evaluation of SAP, routine screening, and tailored regimens personalized by pre-existing patient colonization [146]. Similarly, we also observed that a high MRSA prevalence has led to the adaptation of prophylactic regimens to include vancomycin or entirely change to vancomycin in various surgical fields.

Colonization with antibiotic-resistant pathogens increases the risk for subsequent infections in certain settings [147,148]. A recent systematic review by Righi et al. [21] found a seven-fold higher risk of SSI and post-surgical infections among patients on standard SAP colonized with extended-spectrum cephalosporin-resistant Enterobacterales compared with non-colonized patients. In our review, we observed that pre-existing resistance increased the risk of ARB infections four-fold or more and led to serious infections, while standard prophylaxis was still very effective in preventing bloodstream infections in non-colonized patients [51,113]. For example, due to the changing epidemiology of SSIs in ESBL-colonized patients in elective colorectal surgery, ertapenem prophylaxis was successfully used to reduce infection rates, as an escalation from cephalosporins/metronidazole [59,74,104,131]. This ineffectiveness of prophylactic antibiotics and the potential vicious cycle of escalation of prophylaxis by the need for different or extended regimens has been referred to as “the secondary burden of antibiotic resistance”. Teillant et al. estimated a potential secondary burden of 120,000 additional infections and 6300 infection-related deaths per year for a 30% reduction in efficacy of antibiotic prophylaxis in the USA [149]. Recently, researchers have pinpointed the importance of quantifying this additional burden [150] and broadening the definition of AMR burden [151].

Personalized prophylaxis based on the colonizing resistant pathogen has been suggested as a solution to pre-existing resistance, with alternatives being no prophylaxis, accepting higher infection rates in colonized patients with standard prophylaxis, or using escalated prophylaxis for all patients [152]. Temkin et al. have proposed a framework to guide these decisions in different epidemiological settings based on the number needed to screen to prevent infections [153]. Though promising, it is discomforting to see that universal screening and timely results may not be feasible in large parts of the world. Moreover, a large proportion of unintended cross-overs have been observed even in controlled study settings.

Our study has some limitations. Firstly, due to the heterogeneity, there was a need for detailed subgroup analyses, and only a limited number of studies with comparable information could be grouped together. The surgical studies were quite heterogeneous (e.g., in terms of control group, type of surgery, infection type, causative pathogen, and disease severity) and could have hampered inclusion in the subgroup analysis, thus affecting the precision of estimates. Additional factors in specific subgroups (for example, biofilm formation or other mechanisms of antibiotic resistance development) were not analyzed. Secondly, the background resistance rates could have led to heterogeneity in terms of geographical regions and time periods; however, we do not expect it to confound our findings as we included only studies with a comparison group. Thirdly, almost all our data were derived from high-income countries of the Global North, leaving a knowledge gap, especially for countries with high prevalences of difficult-to-treat Gram-negative bacteria.

## 5. Conclusions

Our systematic review confirms that antibiotic prophylaxis can adequately prevent infections and has beneficial effects on the length of stay in hemato-oncological and surgical settings. However, our findings revealed a significantly increased risk of developing infections caused by bacteria resistant to the antibiotic prophylaxis within the hemato-oncological population, while mortality was not different in patients receiving prophylaxis. This highlights the critical need for meticulous risk assessment when considering prophylaxis in these patients, carefully balancing the benefits of preventing infections against the risk of driving antimicrobial resistance. These insights underscore the importance of developing and implementing targeted antibiotic stewardship programs specifically tailored for hemato-oncological settings to mitigate the emergence of resistance. Surveillance and monitoring of breakthrough infections following antibiotic prophylaxis are increasingly necessary tools to help adapt guidelines in the dynamically evolving prevalence of AMR, especially in the surgical setting. Antibiotic prophylaxis remains a complex trade-off, and further research in this area is inevitably needed to avert the threat of a post-antibiotic era and adapt to changing epidemiological scenarios.

## Figures and Tables

**Table 1:** Characteristics of 109 studies on antibiotic prophylaxis and development of antibiotic resistance, as well as related clinical outcomes, conducted in hemato-oncological (N = 55) and surgical settings (N = 54).

Study Characteristics	Hemato-Oncology (N = 55)	Surgery (N = 54)
Median number of patients included per study (IQR)	210 (104–341)	672 (281–1183)
Year of publication		
1991–2000	10 (18%)	2 (4%)
2001–2010	13 (24%)	13 (24%)
2011–2024	32 (58%)	39 (72%)
Year of start of study period		
1984–2000	21 (38%)	8 (15%)
2001–2010	21 (38%)	29 (54%)
2011–2024	13 (24%)	17 (31%)
Geographical distribution		
Africa	0 (0%)	1 (2%)
Asia	7 (13%)	18 (33%)
Australia	2 (4%)	1 (2%)
Europe	25 (45%)	16 (30%)
North America	14 (25%)	14 (26%)
South America	5 (9%)	0 (0%)
Intercontinental	2 (4%)	4 (7%)
Antibiotic prophylaxis		
Cephalosporin	1 (2%)	24 (44%)
Cephalosporin in combination	0 (0%)	4 (7%)
Quinolone	47 (85%)	9 (17%)
Quinolone in combination	4 (7%)	1 (2%)
Penicillin	0 (0%)	3 (6%)
Penicillin + beta-lactamase inhibitor	0 (0%)	6 (11%)
Carbapenem	0 (0%)	4 (7%)
Others *	3 (5%)	3 (6%)
Study design		
Retrospective cohort study	22 (40%)	17 (32%)
Prospective cohort study	8 (15)	12 (22%)
Randomized controlled trial	8 (15%)	15 (28%)
Prospective before–after study	2 (4%)	1 (2%)
Retrospective before–after study	11 (20%)	4 (7%)
Mixed-cohort study	4 (7%)	2 (4%)
Hemato-oncological treatment		
Stem cell transplantation	18 (33%)	
Chemotherapy	39 (71%)	
Type of surgery	-	
Abdominal surgery		15 (28%)
Trauma surgery		11 (20%)
Urological surgery		12 (22%)
Plastic surgery		2 (4%)
Heart and thoracic surgery		4 (7%)
Gynecological surgery		2 (4%)
Neurosurgery		4 (7%)
Head and neck surgery		3 (6%)
All or unspecific surgeries		1 (2%)
Type of infection **		
All or unspecific infections	39 (71%)	22 (41%)
Bacteremia	48 (87%)	9 (17%)
Surgical site infection	0 (0%)	36 (67%)
Urinary tract infection	1 (2%)	9 (17%)
Abdominal infection	0 (0%)	1 (2%)
Fever/febrile neutropenia	3 (5%)	0 (0%)
Pneumonia	0 (0%)	1 (2%)
Central nervous system	0 (0%)	2 (4%)
Invasive medical device infection	0 (0%)	4 (7%)
Outcome measure ***		
Breakthrough infections (total, including AMR)	45 (82%)	52 (96%)
Antimicrobial resistance in infections	49 (89%)	46 (85%)
Length of hospital stay	8 (15%)	9 (17%)
Mortality	23 (42%)	7 (13%)
Healthcare costs	1 (2%)	3 (6%)

* Others include vancomycin (n = 2) and clindamycin + gentamicin (n = 1) in surgical studies, and fosfomycin (n = 1), trimethoprim/sulfamethoxazole (n = 1), and penicillin + quinolone (n = 1) in hemato-oncological studies; ** IQR, interquartile range; *** one study can provide results for more than one outcome measure/type of infection.

**Table 2:** Pooled estimates of infection rates among patients receiving prophylaxis in comparison with patients not receiving prophylaxis in hemato-oncological studies among subgroups of interest with three or more contributing studies each (n = 35).

Category	N_s_ (N_o_) *	Relative Risk (95% CI)	*p*-Value	I-Square	*p*-Value for Heterogeneity
Reported causative bacteria (as reported in the study)					
All bacteria	27 (42)	0.86 (0.82–0.90)	<0.001	80.0%	<0.001
Causative bacteria classified as Gram-positive/Gram-negative bacteria					
Gram-positive bacteria	10 (10)	1.06 (0.95–1.19)	0.284	78.0%	<0.001
Gram-negative bacteria	16 (16)	0.51 (0.45–0.59)	<0.001	62.0%	<0.001
Type of infection					
All	12 (18)	0.96 (0.90–1.03)	0.279	66.0%	<0.001
Bacteremia	25 (28)	0.81 (0.76–0.87)	<0.001	79.0%	<0.001
Prophylactic antibiotic studied					
Fluoroquinolone	28 (44)	0.86 (0.82–0.90)	<0.001	79.0%	<0.001
Stem cell transplantation					
No	4 (5)	0.71 (0.51–0,99)	0.044	0.0%	0.54
Partly	9 (16)	0.92 (0.86–0.97)	0.005	80.0%	<0.001
Yes	11 (18)	0.75 (0.69–0.81)	<0.001	84.0%	<0.001
Geographical location					
Europe	13 (19)	0.92 (0.86–0.99)	0.024	66.0%	<0.001
North America	8 (15)	0.63 (0.56–0.7)	<0.001	80.0%	<0.001
South America	5 (8)	0.90 (0.83–0.98)	0.017	89.0%	<0.001
Publication year					
1991–2000	6 (10)	0.77 (0.64–0.94)	0.009	73.0%	<0.001
2001–2010	7 (10)	0.93 (0.79–1.08)	0.329	82.0%	<0.001
2011–2024	17 (28)	0.85 (0.81–0.90)	<0.001	80.0%	<0.001

* N_s_ (N_o_) refers to number of studies (number of outcomes).

**Table 3:** Pooled estimates of rates of resistance to prophylactic antibiotics among bacterial infections in patients receiving prophylaxis versus no prophylaxis in hemato-oncological studies (n = 21). Subgroups of interest with three or more contributing studies each are presented.

Category	N_s_ (N_o_) *	Relative Risk (95% CI)	*p*-Value	I-Square	*p*-Value for Heterogeneity
Reported causative bacteria (as reported in the study)					
All bacteria	4 (4)	1.97 (1.02–3.79)	0.044	39.0%	0.178
Enterobacterales	4 (4)	2.93 (2.36–3.63)	<0.001	0.0%	0.598
*Escherichia coli*	9 (11)	1.87 (1.66–2.10)	<0.001	56.0%	0.012
Gram-negative bacteria	7 (9)	2.27 (1.87–2.77)	<0.001	73.0%	<0.001
Gram-positive bacteria	3 (3)	1.01 (0.77–1.31)	0.966	0.0%	0.475
Causative bacteria classified as Gram-positive/Gram-negative bacteria					
Gram-positive bacteria	4 (4)	1.13 (0.87–1.46)	0.353	54.0%	0.091
Gram-negative bacteria	18 (24)	2.14 (1.95–2.34)	<0.001	66.0%	<0.001
Type of infection					
Any or unspecific infections	4 (6)	2.52 (1.62–3.92)	<0.001	65.0%	0.013
bacteremia	17 (19)	2.05 (1.86–2.26)	<0.001	78.0%	<0.001
Prophylactic antibiotic studied					
Fluoroquinolone	19 (23)	2.04 (1.87–2.22)	<0.001	73.0%	<0.001
Stem cell transplantation					
No	3 (3)	2.27 (1.57–3.28)	<0.001	56.0%	0.101
Partly	8 (11)	1.93 (1.73–2.15)	<0.001	56.0%	0.011
Yes	7 (9)	2.54 (2.14–3.01)	<0.001	66.0%	0.003
Geographical location					
Asia	4 (5)	1.29 (1.02–1.63)	0.032	81.0%	<0.001
Europe	9 (11)	2.56 (2.18–3.01)	<0.001	62.0%	0.004
North America	4 (5)	1.86 (1.64–2.11)	<0.001	2.0%	0.396
Publication year					
1991–2000	3 (4)	7.4 (2.77–19.76)	<0.001	19.0%	0.295
2001–2010	5 (6)	2.33 (1.61–3.37)	<0.001	74.0%	0.002
2011–2024	13 (16)	1.98 (1.81–2.16)	<0.001	78.0%	<0.001

* N_s_ (N_o_) refers to number of studies (number of outcomes).

**Table 4:** Overview of studies providing data on colonization with antibiotic-resistant bacteria and risk of infection (n = 7).

Study	Setting	Patients	Patient Groups Being Compared/Antibiotic Prophylaxis	Outcome Studied	Risk of Infection	Unadjusted Relative Risk (95% CI) *
Colonized versus non-colonized patients						
Satlin (2021) [113]	Hemato-oncological	Stem cell transplantation patients	Not colonized/fluoroquinolone (levofloxacin) vs. colonization with fluoroquinolone-resistant Enterobacterales/fluoroquinolone (levofloxacin)	Proportion of BSIs caused by FQ-resistant Gram-negative bacteria	1/80 vs. 16/54	23.3 (3.2–173.5)
Akhmedov (2023) [28]	Hemato-oncological	Stem cell transplantation patients	Colonization with resistant Gram-negative bacteria/no prophylaxis vs. not colonized/no prophylaxis vs. not colonized/fluoroquinolone prophylaxis	General BSI rate	43/147 vs. 9/32 vs. 28/98	1.0 (0.5–1.8) ^§^
Dubinsky-Pertzov (2019) [51]	Surgical	Patients with elective colorectal surgery	Not colonized/cephalosporin + metronidazole vs. colonization with ESBL-producing Enterobacterales/cephalosporin + metronidazole	Proportion of SSIs caused by ESBL-PE	7/440 vs. 16/222	4.5 (1.9–10.9)
Yang (2013) [133]	Surgical	Patients with surgeries in high-risk head and neck cancer patients	Pre-surgical colonization/pre-prophylaxis vs. surgical site infection/clindamycin + gentamicin	Proportion of clindamycin resistance among Gram-positive bacteria	82/171 vs. 26/31	1.75 (1.40–2.18)
Targeted prophylaxis for colonized patients						
Nutman (2020) [104]	Surgical	Patients with elective colorectal surgery	Colonization with ESBL-producing Enterobacterales/adjusted using ertapenem vs. colonization with ESBL-producing Enterobacterales/cephalosporin + metronidazole	Proportion of SSIs caused by ESBL-PE	4/269 vs. 15/209	0.21 (0.07–0.62)
De Pastena (2021) [49]	Surgical	Patients with pancreatic surgery	Colonization with ESBL-producing Enterobacterales/piperacillin–tazobactam vs. colonization with ESBL-producing Enterobacterales/ampicillin–sulbactam	General rate of hospital-acquired infections	11/29 vs. 30/47	0.59 (0.36–0.99)
Newman (2022) [101]	Surgical	Patients with transrectal prostate biopsies	Known colonization status/targeted prophylaxis vs. unknown colonization status/empirical prophylaxis	General BSI rate	9/403 vs. 12/609	1.1 (0.48–2.7)

* Calculated based on reported numbers; ^§^ comparing colonized/no prophylaxis versus non-colonized/no prophylaxis.

## Data Availability

The data were extracted from published articles, and these data, analyzed during the current study, are available from the corresponding author upon reasonable request.

## Supplementary Materials

The following supporting information can be downloaded at https://www.mdpi.com/article/10.3390/antibiotics14090853/s1, Table S1: List of included studies with the main characteristics; Figure S1: Forest plot of the relative risk of resistance rates against prophylactic agent among bacterial infections in hemato-oncological patients receiving prophylaxis as compared to those not receiving prophylaxis reported between 1991–2024, classified by country of study (N = 21); Figure S2: Forest plot of the relative risk of resistance rates against prophylactic agent among bacterial infections in hemato-oncological patients receiving prophylaxis as compared to those not receiving prophylaxis reported between 1991–2024, classified by year of publication (N = 21); Table S2: Pooled estimates of resistance rates among causative pathogens with any antibiotic resistance in comparison with patients not receiving prophylaxis in hemato-oncological studies among subgroups of interest with three or more contributing studies each (N = 21); Figure S3: Forest plot of the weighted mean difference of length of hospital stay between hemato-oncological patients receiving prophylaxis as compared to those not receiving prophylaxis reported between 1991–2024 (N = 5); Figure S4: Forest plot of the relative risk of surgical site infections between surgical patients receiving prophylaxis as compared to those not receiving prophylaxis reported between 1991–2024 (N = 5); Figure S5: Forest plot of the relative risk of any infection among surgical patients receiving short prophylaxis as compared to those on extended prophylaxis reported between 1991–2024 (N = 14); Figure S6: Risk of bias assessment of the included hemato-oncological studies with a comparison group using modified Joanna Briggs Institute’s critical appraisal tool (N = 55); Figure S7: Summary of the risk of bias assessment of the included hemato-oncological studies with a comparison group using modified Joanna Briggs Institute’s critical appraisal tool (N = 55); Figure S8: Risk of bias assessment of the included surgical studies with a comparison group using modified Joanna Briggs Institute’s critical appraisal tool (N = 54); Figure S9: Summary of the risk of bias assessment of the included surgical studies with a comparison group using modified Joanna Briggs Institute’s critical appraisal tool (N = 54).

## Abbreviations

LOS	Length of Stay
AMR	Antimicrobial Resistance
GDP	Gross Domestic Product
ARB	Antibiotic-Resistant Bacteria
WMD	Weighted Mean Difference
FQRE	Fluoroquinolone-Resistant Enterobacterales
ESBL	Extended-Spectrum Beta-Lactamase
ESBL-PE	ESBL-Producing Enterobacterales
MRSA	Methicillin-Resistant *Staphylococcus aureus*
RR	Relative Risk
CI	Confidence Interval
SSI	Surgical-Site Infection
ASHP	American Society of Health-System Pharmacists
EAU	European Association of Urology
TRPB	Transrectal Prostate Biopsy
DGHO	Deutsche Gesellschaft für Hämatoonkologie/German Association of Hemato-Oncology
ASCO	American Society of Clinical Oncology
IDSA	Infectious Diseases Society of America
