# Supplementary material for: The Impact of Antibiotic Prophylaxis on Antibiotic Resistance, Clinical Outcomes, and Costs in Adult Hemato-Oncological and Surgical Patients: A Systematic Review and Meta-Analysis
Source: Antibiotics (Basel). 2025 Aug 22;14(9):853. doi: 10.3390/antibiotics14090853 (PMC12466468; doi:10.3390/antibiotics14090853)
Supplement: Supplementary file 1 [file antibiotics-14-00853-s001.zip › antibiotics-3789573-supplementary.pdf]

Supplementary material

The Impact of Antibiotic Prophylaxis on Antibiotic Resistance, Clinical Outcomes, and Costs in adult Hemato-Oncological and Surgical Patients: A Systematic Review and Meta-Analysis

Marissa Rink, Beryl Primrose Gladstone, Lea Ann Nikolai, Michael Bitzer, Evelina Tacconelli, Siri Göpel

Table of contents

PRISMA checklist 3

Supplementary section 1: Search terms 6

Supplementary section 2: Descriptive results 7

Supplementary Table 1: List of included studies with the main characteristics 8

Supplementary Figure 1: Forest plot of the relative risk of resistance rates against prophylactic agent among bacterial infections in hemato-oncological patients receiving prophylaxis as compared to those not receiving prophylaxis reported between 1991-2024, classified by country of study (N=21) 30

Supplementary Figure 2: Forest plot of the relative risk of resistance rates against prophylactic agent among bacterial infections in hemato-oncological patients receiving prophylaxis as compared to those not receiving prophylaxis reported between 1991-2024, classified by year of publication (N=21) 31

Supplementary Table 2: Pooled estimates of resistance rates among causative pathogens with any antibiotic resistance in comparison with patients not receiving prophylaxis in hemato-oncological studies among subgroups of interest with three or more contributing studies each (N=21) 32

Supplementary Figure 3: Forest plot of the weighted mean difference of length of hospital stay between hemato-oncological patients receiving prophylaxis as compared to those not receiving prophylaxis reported between 1991-2024 (N=5) 33

Supplementary Figure 4: Forest plot of the relative risk of surgical site infections between surgical patients receiving prophylaxis as compared to those not receiving prophylaxis reported between 1991-2024 (N=5) 34

Supplementary Figure 5: Forest plot of the relative risk of any infection among surgical patients receiving short prophylaxis as compared to those on extended prophylaxis reported between 1991-2024 (N=14) 35

Supplementary Figure 6: Risk of bias assessment of the included hemato-oncological studies with a comparison group using modified Joanna Briggs Institute's critical appraisal tool (N=55) 36

Supplementary Figure 7: Summary of the risk of bias assessment of the included hemato-oncological studies with a comparison group using modified Joanna Briggs Institute’s critical appraisal tool (N=55) 38

Supplementary Figure 8: Risk of bias assessment of the included surgical studies with a comparison group using modified Joanna Briggs Institute’s critical appraisal tool (N=54) 39

40

Supplementary Figure 9: Summary of the risk of bias assessment of the included surgical studies with a comparison group using modified Joanna Briggs Institute’s critical appraisal tool (N=54) 41

References 42

PRISMA checklist

Section and Topic	Item #	Checklist item	Location where item is reported
**TITLE**			Page numbers
Title	1	Identify the report as a systematic review.	1
**ABSTRACT**			
Abstract	2	See the PRISMA 2020 for Abstracts checklist.	2
**INTRODUCTION**			
Rationale	3	Describe the rationale for the review in the context of existing knowledge.	3
Objectives	4	Provide an explicit statement of the objective(s) or question(s) the review addresses.	3
**METHODS**			
Eligibility criteria	5	Specify the inclusion and exclusion criteria for the review and how studies were grouped for the syntheses.	4
Information sources	6	Specify all databases, registers, websites, organisations, reference lists and other sources searched or consulted to identify studies. Specify the date when each source was last searched or consulted.	4
Search strategy	7	Present the full search strategies for all databases, registers and websites, including any filters and limits used.	4
Selection process	8	Specify the methods used to decide whether a study met the inclusion criteria of the review, including how many reviewers screened each record and each report retrieved, whether they worked independently, and if applicable, details of automation tools used in the process.	4
Data collection process	9	Specify the methods used to collect data from reports, including how many reviewers collected data from each report, whether they worked independently, any processes for obtaining or confirming data from study investigators, and if applicable, details of automation tools used in the process.	4
Data items	10a	List and define all outcomes for which data were sought. Specify whether all results that were compatible with each outcome domain in each study were sought (e.g. for all measures, time points, analyses), and if not, the methods used to decide which results to collect.	4
	10b	List and define all other variables for which data were sought (e.g. participant and intervention characteristics, funding sources). Describe any assumptions made about any missing or unclear information.	4
Study risk of bias assessment	11	Specify the methods used to assess risk of bias in the included studies, including details of the tool(s) used, how many reviewers assessed each study and whether they worked independently, and if applicable, details of automation tools used in the process.	5
Effect measures	12	Specify for each outcome the effect measure(s) (e.g. risk ratio, mean difference) used in the synthesis or presentation of results.	4
Synthesis methods	13a	Describe the processes used to decide which studies were eligible for each synthesis (e.g. tabulating the study intervention characteristics and comparing against the planned groups for each synthesis (item #5)).	4
	13b	Describe any methods required to prepare the data for presentation or synthesis, such as handling of missing summary statistics, or data conversions.	4

Section and Topic	Item #	Checklist item	Location where item is reported
	13c	Describe any methods used to tabulate or visually display results of individual studies and syntheses.	4
	13d	Describe any methods used to synthesize results and provide a rationale for the choice(s). If meta-analysis was performed, describe the model(s), method(s) to identify the presence and extent of statistical heterogeneity, and software package(s) used.	4, 5
	13e	Describe any methods used to explore possible causes of heterogeneity among study results (e.g. subgroup analysis, meta-regression).	5
	13f	Describe any sensitivity analyses conducted to assess robustness of the synthesized results.	5
Reporting bias assessment	14	Describe any methods used to assess risk of bias due to missing results in a synthesis (arising from reporting biases).	5
Certainty assessment	15	Describe any methods used to assess certainty (or confidence) in the body of evidence for an outcome.	5
**RESULTS**			
Study selection	16a	Describe the results of the search and selection process, from the number of records identified in the search to the number of studies included in the review, ideally using a flow diagram.	5, 6
	16b	Cite studies that might appear to meet the inclusion criteria, but which were excluded, and explain why they were excluded.	6 (flowchart)
Study characteristics	17	Cite each included study and present its characteristics.	Suppl p.5-26
Risk of bias in studies	18	Present assessments of risk of bias for each included study.	Suppl. p.33-34, 36-37
Results of individual studies	19	For all outcomes, present, for each study: (a) summary statistics for each group (where appropriate) and (b) an effect estimate and its precision (e.g. confidence/credible interval), ideally using structured tables or plots.	P 6-18 Suppl p.27-32
Results of syntheses	20a	For each synthesis, briefly summarise the characteristics and risk of bias among contributing studies.	p 6-18 Suppl 33-34
	20b	Present results of all statistical syntheses conducted. If meta-analysis was done, present for each the summary estimate and its precision (e.g. confidence/credible interval) and measures of statistical heterogeneity. If comparing groups, describe the direction of the effect.	P 6-18
	20c	Present results of all investigations of possible causes of heterogeneity among study results.	P 6-18
	20d	Present results of all sensitivity analyses conducted to assess the robustness of the synthesized results.	P 6-18
Reporting biases	21	Present assessments of risk of bias due to missing results (arising from reporting biases) for each synthesis assessed.	18
Certainty of evidence	22	Present assessments of certainty (or confidence) in the body of evidence for each outcome assessed.	P 6-18
**DISCUSSION**			
Discussion	23a	Provide a general interpretation of the results in the context of other evidence.	18
	23b	Discuss any limitations of the evidence included in the review.	20

Section and Topic	Item #	Checklist item	Location where item is reported
	23c	Discuss any limitations of the review processes used.	20
	23d	Discuss implications of the results for practice, policy, and future research.	21
**OTHER INFORMATION**			
Registration and protocol	24a	Provide registration information for the review, including register name and registration number, or state that the review was not registered.	4
	24b	Indicate where the review protocol can be accessed, or state that a protocol was not prepared.	4
	24c	Describe and explain any amendments to information provided at registration or in the protocol.	4
Support	25	Describe sources of financial or non-financial support for the review, and the role of the funders or sponsors in the review.	22
Competing interests	26	Declare any competing interests of review authors.	22
Availability of data, code and other materials	27	Report which of the following are publicly available and where they can be found: template data collection forms; data extracted from included studies; data used for all analyses; analytic code; any other materials used in the review.	22

Supplementary section 1: Search terms

Prophylaxis and resistance development

("resistant""[tw] OR ""resistance""[tw] OR "resistant""[MeSH] OR ""resistance""[MeSH] OR ESBL[tw] OR ""Extended spectrum beta-lactamase""[tw] OR ESBL[Mesh] OR ""Extended spectrum beta-lactamase"" [Mesh]) OR "Extended spectrum ? lactamase"[tw] OR "Extended spectrum ? lactamase"[Mesh] OR carbapenem[mesh] OR carbapenem[tw] OR carbapenemase[Mesh] OR carbapenemase[tw] OR resistan[tw]) OR resistance[Mesh]) AND (Prophylac*[tw] OR prophylaxis [MeSH] OR prophylaxis [tw])*

Outcomes

A. incidence

Incidence[tw] OR colonization[tw] OR prevalence[tw] OR Incidence[MeSH] OR colonization[MeSH] OR prevalence[MeSH]

B. mortality, cost or length of hospital stay

(length of stay[MeSH] OR (hospitalisation[tw] AND length[tw]) OR (hospitalization[tw] AND length[tw]) OR "length of hospitalisation"[tw] OR "length of hospitalization"[tw] OR "duration of hospitalization"[tw] OR "duration of hospitalization"[tw] OR LOS[tw] OR (period[tw] OR length[tw]) AND (hospital stay[tw] OR hospitalization[tw] OR hospitalization[tw])) OR (mortality[MeSH] OR mortality[tw] OR death rate[tw] OR fatality[tw] OR survival rate[tw] OR death[tw] OR died[tw] OR dead[tw]) OR (cost[Title/Abstract] OR "costs and cost analysis"[MeSH:noexp])*

Study setting

A. perioperative antimicrobial prophylaxis in surgical setting

First search: "perioperative"[Text Word] OR "periopera*"[Text Word] OR "peri operativ*"[Text Word] OR "one-*shot"[tw] OR "one shot"[tw] OR "single-shot"[tw] OR "single shot"[tw] OR peri-operativ*[tw] OR "surgical prophylaxis"[tw]*

Additional search: *(((cohort*[TiAb] AND study*[TiAb]) OR observational study*[TiAb] OR controlled before-and-after study*[TiAb] OR CBAs[TiAb] OR interrupted time series[TiAb] OR ITS[TiAb]) AND (antibiotic*[TiAb] OR antimicrobial*[TiAb] OR antiinf*[TiAb]) AND (prophyla*[TiAb] OR preventive*[TiAb] OR prevention*[TiAb] OR preventing[TiAb] OR premedication[TiAb]) AND (surg*[TiAb]))*

B. antimicrobial prophylaxis in hemato-oncological setting

Erste Suche: *neutropenia[Text Word] OR neutropenia[MeSH] OR "neutropeni*"[tw] OR neutropenic[tw] OR "medical oncology"[Text Word] OR "immuno-suppression"[Text Word] OR "immuno-compromised"[Text Word] OR "immuno-suppress*"[Text Word] OR "immuno suppress*"[tw] OR immunosuppress*[tw] OR hematology[Text Word] OR "medical oncology"[MeSH Terms] OR hematology[MeSH Terms] OR hemato*[tw] OR hemato*[MeSH] OR haemato*[tw] OR immunocompromis*[tw] OR immuno-compromis*[tw] OR "immuno compromis*"[tw] OR "immune compromis*"[tw] OR "immune suppress*"[tw]*

Additional search: *(((cohort*[TiAb] AND study*[TiAb]) OR observational study*[TiAb] OR controlled before-and-after study*[TiAb] OR CBAs[TiAb] OR interrupted time series[TiAb] OR ITS[TiAb]) AND (antibiotic*[TiAb] OR antimicrobial*[TiAb] OR antiinf*[TiAb]) AND (prophyla*[TiAb] OR preventive*[TiAb] OR prevention*[TiAb] OR preventing[TiAb] OR premedication[TiAb])*

Supplementary section 2: Descriptive results

In hemato-oncological settings, quinolones were compared with other quinolones or with combination prophylaxis including a quinolone (9, 16.4%). In surgical settings, cephalosporins were primarily compared with other cephalosporins, either as monotherapy or in combination with another antibiotic (16, 29.6%). In eight studies (14.8%) a cephalosporin was compared with another antibiotic. Five studies (9.3%) compared carbapenems primarily with second-generation cephalosporins alone or in combination. Perioperative SAP was studied in 40 (59.8%) studies, prolonged SAP in 12 (22.2%) and the duration was not clearly reported in 2 studies (3.7%). The SAP prolongation was based on treatment protocols or guidelines (5, 41.7%). There was no justification reported in 7 studies (58.3%), of which 3 were urological surgeries, 2 abdominal surgeries, 1 neurosurgery and 1 head and neck surgery.

Surgical studies – supplementary information:

Three studies compared prophylactic regimens in urological surgery patients: quinolone was standard prophylaxis compared to an effective alternative - Pivmecillinam and amoxicillin/clavulanic acid combination, Bactrim DS and Gentamicin combination, and third-generation cephalosporin.

Supplementary Table S1: List of included studies with the main characteristics

First author	Study period	Study design	Patient population	Country	Main objective	Outcomes	Prophylaxis class
**Studies in the hemato-oncological setting**							
Signorelli (2020) (2)	2013-2016	Retrospective before after study	Hemato-oncological	USA	To investigate risks and benefits of prophylaxis in stem cell transplantation	antimicrobial resistance, infection (BSI)	no prophylaxis vs. Fluoroquinolone
Craig M (2007) (3)	2002-2004	Mixed (Prosp. & Retrosp.) cohort study	Hemato-oncological	USA	To evaluate the use of prophylaxis during neutropenia	antimicrobial resistance, infection (BSI), mortality	Fluoroquinolone vs. no prophylaxis
Zaidi (2001) (4)	1996-1997	Retrospective cohort study	Hemato-oncological	UK, Pakistan	To investigate benefits and resistance development under prophylaxis	antimicrobial resistance, infection (BSI)	Fluoroquinolone vs. Fluoroquinolone
Gomez (2003) (5)	1993-1996	Retrospective before after study	Leukemia	Spain	To evaluate the effects of prophylaxis on incidence of sepsis and patterns of resistance	antimicrobial resistance, infection (BSI)	no prophylaxis vs. Fluoroquinolone
Timmers (2007) (6)	2002-2005	RCT	Hemato-oncological	Netherlands	To compare two different prophylactic regimens and to monitor emerging antimicrobial resistance	antimicrobial resistance, infection (BSI)	Fluoroquinolone vs. Fluoroquinolone + Penicillin
Chong (2011) (7)	2003-2009	Retrospective before after study	Hemato-oncological	Japan	To assess the effects of prophylaxis on etiology and antibiotic resistance patterns of blood culture isolates	antimicrobial resistance, infection (BSI), mortality	Fluoroquinolone vs. no prophylaxis

Wolska (2012) (8)	2005-2008	Retrospective cohort study	Hemato-oncological	Poland	To evaluate the efficacy of prophylaxis in the reduction of infectious incidents	antimicrobial resistance, infection (BSI), LOS	no prophylaxis vs. Fluoroquinolone
Cattaneo (2008) (9)	2004-2005	Prospective cohort study	Hemato-oncological	Italy	To disclose evolving trends in the epidemiology of infections and emerging antibiotic resistance	antimicrobial resistance, infection (BSI)	no prophylaxis vs. Fluoroquinolone
Carratala (1995) (10)	1988-1992	Retrospective cohort study	Hemato-oncological	Spain	To identify incidence and risk factors for quinolone resistant bacteremia	antimicrobial resistance, infection (BSI)	Fluoroquinolone vs. no prophylaxis
Hakki (2019) (11)	2012-2018	Retrospective cohort study	Hemato-oncological	USA	To investigate the impact of prophylaxis on emergence of resistance	antimicrobial resistance, infection (BSI)	Fluoroquinolone vs. Non-Fluoroquinolone
Lee (2018) (13)	2006-2013	Retrospective cohort study	Leukemia	UK	To evaluate the efficacy and safety of prophylactic oral levofloxacin	antimicrobial resistance, infection	Fluoroquinolone vs. no prophylaxis
Kern (2018) (14)	2009-2014	Prospective cohort study	Hemato-oncological	Germany, Austria, Switzerland	To describe the effect of emerging flourguinolone resistance on the efficacy of prophylaxis	antimicrobial resistance, infection (BSI), mortality	Fluoroquinolone vs. no prophylaxis
Hauck (2018) (17)	2000-2015	Retrospective cohort study	Hemato-oncological	USA	To evaluate the trend of fluoroquinolone non-susceptibility rates in E. coli isolates from patients' blood and urine cultures	antimicrobial resistance, infection (BSI, UTI)	Fluoroquinolone vs. no prophylaxis

Engels (1999) (19)	1995-1996	Retrospective cohort study	Hemato-oncological	USA	To improve the understanding of determinants of infection risk for transplant recipients with focus on antimicrobial administration	antimicrobial resistance, infection	Fluoroquinolone + Cotrimoxazole vs. no prophylaxis
Gudiol (2013) (20)	1991-2010	Prospective before after study	Hemato-oncological	Spain	To identify the impact of antibacterial prophylaxis on the incidence and etiology of blood stream infections	antimicrobial resistance, infection (BSI), mortality	Fluoroquinolone vs. no prophylaxis
Hsueh (2005) (22)	2001-2003	Retrospective before after study	Hemato-oncological	Taiwan	To assess the relationship between fluoroquinolone use and drug resistance	antimicrobial resistance, infection	Fluoroquinolone vs. Fluoroquinolone
Ward (1993) (23)	1984-1986	RCT	Leukemia	USA	To evaluate the efficacy and safety of oral TMP-SMZ used prophylactically in adult patients with acute leukemia	antimicrobial resistance, infection, mortality, LOS	Cotrimoxazole vs. no prophylaxis
Cattaneo (2016) (24)	2012-2014	Prospective cohort study	Leukemia	Italy	To describe the specific epidemiological scenario of bacterial infections and antibiotic resistance in acute leukemia patients during treatment, often affected by severe neutropenia	antimicrobial resistance, infection (BSI)	Fluoroquinolone vs. no prophylaxis
Cumpston (2013) (29)	1999-2009	Mixed (Prosp. & Retrosp.) cohort study	Hemato-oncological	USA	To assess the efficacy of long-term cycling antibiotics in stem cell transplant patients	antimicrobial resistance, infection (BSI)	Fluoroquinolone vs. Fluoroquinolone vs. no prophylaxis

Ganti (2017) (31)	2006-2015	Retrospective cohort study	Leukemia	USA	To evaluate the impact of antibacterial prophylaxis on clinical outcomes and complications before and after protocol implementation.	antimicrobial resistance, infection, mortality	Fluoroquinolone vs. no prophylaxis
Kern (2005) (33)	1992-1999	Retrospective before after study	Hemato-oncological	Germany	To assess the impact of fluoroquinolone prophylaxis on the epidemiologic evolution of fluoroquinolone resistance of E. coli clinical isolates	antimicrobial resistance, infection	Fluoroquinolone vs. no prophylaxis vs. Fluoroquinolone
Prentice (2001) (35)	1987-1999	RCT	Hemato-oncological	UK	To assess the risk of emergence of resistant organisms after the implementation of a certain protocol	antimicrobial resistance, infection	Fluoroquinolone vs. Neomycin
Satlin (2015) (36)	2003-2010	Retrospective cohort study	Hemato-oncological	USA	To assess the incidence of bloodstream infection (BSI) and febrile neutropenia within 30 days of transplantation in patients receiving or not receiving fluoroquinolone prophylaxis	antimicrobial resistance, infection (BSI)	Fluoroquinolone vs. no prophylaxis
Simondsen (2013)(37)	2008-2011	Retrospective before after study	Hemato-oncological	USA	To assess the effect of fluoroquinolone prophylaxis on the incidence of neutropenic fever, fluoroquinolone resistance, C. difficile-associated diarrhea nd MRSA infections	antimicrobial resistance, infection, LOS	no prophylaxis vs. Fluoroquinolone
Zavrelova (2019) (38)	2013-2015	Retrospective cohort study	Myeloma	Czech Republic	To evaluate the impact of cessation of ciprofloxacin prophylaxis during stem cell transplantation for multiple myeloma	antimicrobial resistance, infection (BSI), mortality	Fluoroquinolone vs. no prophylaxis

Averbuch (2017) (40)	2014-2015	Prospective cohort study	Hemato-oncological	Multi-national	To describe resistance rates and risk factors in Gram negative rods bacteremia in stem cell transplant patients	antimicrobial resistance, infection	Fluoroquinolone vs. no prophylaxis
Verlinden (2014) (41)	2009-2011	Retrospective before after study	Hemato-oncological	Belgium	To assess the impact of fluoroquinolone prophylaxis on infection rate and resistance	antimicrobial resistance, infection (BSI), mortality	Fluoroquinolone + Cotrimoxazole vs. no prophylaxis
Sohn (2012) (42)	2001-2008	Retrospective cohort study	Hemato-oncological	South Korea	To investigate the efficacy of antibiotic prophylaxis in patients undergoing autologous stem cell transplantation for multiple myeloma and non-Hodgkin lymphoma	antimicrobial resistance, infection	Fluoroquinolone vs. no prophylaxis
Macesic (2014) (43)	2001-2010	Retrospective before after study	Hemato-oncological	Australia	To determine the microbial epidemiology and trends in susceptibility of organisms cultured from patients after stem cell transplantation and to assess the independent predictors of mortality for bloodstream infection (BSI) in this patient population	antimicrobial resistance, infection (BSI)	Fluoroquinolone vs. no prophylaxis
Saito (2008) (45)	2001-2005	Retrospective before after study	Hemato-oncological	Japan	To assess the effects of restricted prophylactic use of fluoroquinolone on the spectrum of infectious organisms and patterns of antibiotic resistance in patients with bloodstream infection	antimicrobial resistance, infection (BSI)	Fluoroquinolone vs. no prophylaxis

Schroeder (1992) (46)	1987-1988	RCT	Solid tumors	Germany	To assess the impact of ofloxacin prophylaxis in neutropenic patients on the incidence and severity of infections and cost effectiveness	antimicrobial resistance, infection, mortality	Fluoroquinolone vs. no prophylaxis
Guiot (1992) (47)	1988-1990	RCT	Hemato-oncological	Netherlands	To compare the ability of penicillin G and cotrimoxazole to prevent streptococcal septicemia in patients receiving aggressive antileukemic therapy and to evaluate their other properties	antimicrobial resistance, infection	Penicillin vs. Cotrimoxazole
Trecarichi (2019) (48)	2016-2017	Prospective cohort study	Hemato-oncological	Italy	To identify risk factors for third generation cephalosporin resistance in E. coli and prognostic factors of resistant blood stream infections	antimicrobial resistance, infection (BSI)	Fluoroquinolone vs. no prophylaxis
Munoz (1999) (49)	1994-1997	Mixed (Prosp. & Retrosp.) cohort study	Lymphoma	Spain	To analyze the impact of intensified prophylaxis with ofloxacin plus rifampin on infectiou morbidity compared with the efficacy of either norfloxacin or no prophylaxis	antimicrobial resistance, infection (BSI), LOS	Fluoroquinolone + Rifamy vs. Fluoroquinolone
Martino (1998) (51)	1995-1996	Retrospective cohort study	Hemato-oncological	Spain	To analyze the impact of prophylaxis on infectious morbidity	antimicrobial resistance, infection (BSI)	Fluoroquinolone vs. no prophylaxis
Lee (2002) (54)	1999-1999	RCT	Leukemia	Korea	To access the effectiveness of antimicrobial prophylaxis with orally absorbable antibiotics	antimicrobial resistance, infection, LOS, mortality	Fluoroquinolone + Macrol vs. no prophylaxis

Garnica (2013) (55)	2005-2008	Retrospective before after study	Hemato-oncological	Brazil	To evaluate the impact of quinolone prophylaxis given during neutropenia on different outcomes, with special attention to the rates of resistance	antimicrobial resistance, infection (BSI), LOS	Fluoroquinolone vs. no prophylaxis
D'Antonio (1994) (56)	-	RCT	Hemato-oncological	Italy	To compare the efficacy of different fluoroquinolones in preventing bacterial infections in neutropenic patients	antimicrobial resistance, infection (BSI), LOS	Fluoroquinolone vs. Fluoroquinolone vs. Fluoroquinolone
Nucci (1994) (57)	1989-1993	Retrospective cohort study	Leukemia	Brazil	To assess the use of quinolones in the prophylaxis of bacterial infections in neutropenic patients treated for acute non-lymphocytic leukemia and report the patterns of resistance of Gram-negative strains to these agents	antimicrobial resistance, infection (BSI), mortality	Fluoroquinolone vs. no prophylaxis
Carena (2016) (58)	1997-2014	Prospective cohort study	Hemato-oncological	Argentinia	To describe the clinical, microbiological and therapeutic characteristics, and outcome of patients in the first episode of febrile neutropenia, comparing those who received levofloxacin prophylaxis with those who didn't.	antimicrobial resistance, infection (BSI)	Fluoroquinolone vs. no prophylaxis
Delarive (2000) (59)	1993-1995	Retrospective cohort study	Hemato-oncological	Switzerland	To assess the effect of antibiotic prophylaxis with ciprofloxacin and penicillin on the prevention of bacterial infections	antimicrobial resistance, infection (BSI)	Penicillin + Fluoroquinolone vs. no prophylaxis

Slavin (2007) (60)	-	RCT	Hemato-oncological	Australia	To assess the impact of antibiotic prophylaxis on the occurrence of fever, bacterial infection and the need for antibiotic usage	antimicrobial resistance, infection (BSI), mortality, LOS	4GCephalosporin + 4GCephalosporin vs. no prophylaxis
Mahida (2018) (61)	2000-2016	Mixed (Prosp. & Retrosp.) cohort study	Hemato-oncological	UK	To observe the association between administration of flouroquinolone prophylaxis and the development of resistance	antimicrobial resistance, infection (BSI)	Fluoroquinolone vs. no prophylaxis
Ugarte-Torres (2006) (62)	2000-2003	Retrospective cohort study	Leukemia	Mexico	To describe the usefulness of fluoroquinolones in the prevention of bacteraemias caused by gram-negative germs in neutropenic patients with acute leukaemia, as well as their impact on mortality and on the development of bacteraemias caused by resistant strains, in a hospital with a high rate of resistance to fluoroquinolones	antimicrobial resistance, infection (BSI), mortality	Fluoroquinolone vs. no prophylaxis
Sojo (2016) (63)	2006-2010	Retrospective cohort study	Hemato-oncological	Spain	To assess the clinical utility and to eliminate the impact on the frequency and type of infections that the use of levofloxacin has had as antibacterial prophylaxis in recipients of stem cell transplantation.	antimicrobial resistance, infection, mortality	Fluoroquinolone vs. no prophylaxis
Qin (2007) (64)	-	Retrospective cohort study	Leukemia	China	To investigate the clinical benefits and the impacts on distribution and antibiotic resistance of pathogenic bacterium associated with	antimicrobial resistance, infection	Fluoroquinolone vs. no prophylaxis

					fluoroquinolone prophylaxis during neutropenia in patients with acute leukemia		
Sinko (2011) (65)	2008-2009	Retrospective before after study	Hemato-oncological	Hungary	To study the effect of fluoroquinolone prophylaxis on Gram-negative bacteremia	antimicrobial resistance, infection (BSI)	Fluoroquinolone vs. no prophylaxis
Caro (2019) (68)	2012-2016	Retrospective cohort study	Leukemia	USA	To evaulate the efficacy of fluoroquinolone prophylaxis in a population with high rates of fluoroquinolone resistance	antimicrobial resistance, infection	Fluoroquinolone vs. no prophylaxis
Satlin (2021) (71)	2016-2019	Prospective cohort study	Hemato-oncological	USA	To determine the prevalence of FQRE colonization on admission for HCT, to compare the risk of gram-negative BSI in patients with and without pretransplant FQRE colonization and to compare the genetic relatedness between colonizing and bloodstream FQRE isolates among bacteremic patients	antimicrobial resistance, infection (BSI), mortality	Fluoroquinolone vs. Fluoroquinolone
Clerici (2023) (73)	2018-2020	Prospective cohort study	Hemato-oncological	Italy	To analyze any changes in pre-engraftment bloodstream infections incidence and AMR of GNB, febrile neutropenia and mortality in stem cell transplant recipients.	antimicrobial resistance, infection (BSI), mortality	Fluoroquinolone vs. no prophylaxis

Guare (2024) (74)	2016-2021	Retrospective cohort study	Myeloma	USA	To determine the effect of bacterial prophylaxis on neutropenic fever and bacteremia within 30 days of ASCT	antimicrobial resistance, infection (BSI)	Fluoroquinolone vs. Fluoroquinolone + Doxycycline
Urbino (2023) (75)	2001-2019	Retrospective cohort study	Leukemia	Italy	To evaluate the impact of avoiding antibacterial prophylaxis on infections and early mortality rates in AML patients during post-induction aplasia.	antimicrobial resistance, infection (BSI), mortality	Fluoroquinolone vs. no prophylaxis
Guimaraes (2022) (76)	2016-2018	Prospective before after study	Hemato-oncological	Brazil	To investigate whether avoiding fluoroquinolone prophylaxis during neutropenia reduces antibiotic resistance in Gram-negative bloodstream infections and ist impact on BSI-related mortality in HSCT patients.	antimicrobial resistance, infection (BSI), mortality	Fluoroquinolone vs. no prophylaxis
Caro (2022) (77)	2012-2019	Retrospective cohort study	Leukemia	USA	To evaluate the impact of antimicrobial prophylaxis on the development of neutropenic fever, mortality, systemic infections, and antibiotic resistance in AML patients.	antimicrobial resistance, infection (BSI), mortality	Fluoroquinolone vs. no prophylaxis
Akhmedov (2023) (81)	2018-2021	Retrospective cohort study	Hemato-oncological	Russia	To analyze the impact of fluoroquinolone prophylaxis and sequential therapy on pre-engraftment BSI risk and etiology in allo-HCT recipients, considering gut colonization with resistant Gram-negative bacteria.	antimicrobial resistance, infection (BSI), mortality	no prophylaxis vs. no prophylaxis vs. Fluoroquinolone

**Studies in the surgical setting**							
Antsupova (2014) (1)	2010-2013	Retrospective cohort study	Urological surgery	Denmark	To evaluate the prevalence of post-TRUBP infections and the antimicrobial resistance after a change in antibiotic prophylaxis	antimicrobial resistance, infection (BSI)	Fluoroquinolone vs. Penicillin with Beta-Lactamase Inhibitor + Penicillin vs. Penicillin with Beta-Lactamase Inhibitor + Penicillin
Korinek (2006) (12)	1997-2003	Prospective cohort study	Craniotomy and neurosurgery	France	To evaluate incidence and risk factors of postoperative meningitis, with special emphasis on antibiotic prophylaxis	antimicrobial resistance, infection (SSI), mortality	Beta-Lactam antibiotics vs. no prophylaxis
Goldstein (2009) (15)	-	RCT	Abdominal surgery	USA	To compare the efficacy of Ertapenem and Cefotetan in elective colorectal surgery	antimicrobial resistance, infection	Carbapenem vs. Second-Generation Cephalosporin
Lee (2016) (16)	2003-2013	Retrospective cohort study	Urological surgery	Korea	To evaluate various clinical factors including antimicrobial regimens associated with infectious complication after transrectal ultrasound-guided prostate biopsy.	antimicrobial resistance, infection, mortality	Fluoroquinolone vs. Third-Generation Cephalosporin
Itani (2006) (18)	2002-2005	RCT	Abdominal surgery	USA	To assess the efficacy and safety of antibiotic prophylaxis with ertapenem, as compared with cefotetan, in patients undergoing elective colorectal surgery.	antimicrobial resistance, infection (SSI)	Carbapenem vs. Second-Generation Cephalosporin

Harbarth (2000) (21)	1993-1997	Prospective cohort study	Heart and thoracic surgery	USA	To examine the effect of prolonged antibacterial prophylaxis on the risk of SSI and the selection of antibiotic-resistant microorganisms after coronary artery bypass graft surgery	antimicrobial resistance, infection (SSI)	First-Generation Cephalosporin + First-Generation Cephalosporin vs. First-Generation Cephalosporin + First-Generation Cephalosporin
Adibi (2013) (25)	2010-2011	Mixed (Prosp. & Retrosp.) cohort study	Urological surgery	USA	To evaluate the incidence of infectious complications requiring hospitalization after transrectal ultrasound guided prostate biopsy, comparing an augmented regimen of antibiotic prophylaxis to the standard regimen, and established cost-effectiveness	antimicrobial resistance, infection (SSI)	Fluoroquinolone vs. Fluoroquinolone + Gentamicin
Cohen (2017) (26)	2008-2016	Retrospective cohort study	All elective surgeries	USA	To assess the relationship between use of surgical antibiotic prophylaxis and development of post-operative antibiotic-resistant infections	antimicrobial resistance, infection	Cephalosporin vs. no prophylaxis
Merrer (2006) (27)	2004-2005	Prospective cohort study	Trauma surgery	France	To assess the impact of antibiotic prophylaxis on the emergence of vancomycin-resistant bacteria and the incidence of surgical site infection after vancomycin or cefazolin prophylaxis for femoral neck fracture surgery	antimicrobial resistance, infection (SSI), mortality	Vancomycin vs. First-Generation Cephalosporin

Cammann (2016) (28)	2001-2010	Retrospective before after study	Abdominal surgery	Germany	To investigate the influence of perioperative antibiotic prophylaxis on the postoperative course of cholangitis after hepatobiliary surgery depending on the choice of intraoperative antibiotic prophylaxis	antimicrobial resistance, infection, mortality, LOS	Penicillin with Beta-Lactamase Inhibitor vs. Fluoroquinolone + Metronidazole
Fong (2016) (30)	2008-2013	Retrospective cohort study	Abdominal surgery	USA, Italy	To analyze the microbiology of post-PD wound infection cultures and the effectiveness of institution-based perioperative antibiotic protocols	antimicrobial resistance, infection (SSI)	Second-Generation Cephalosporin vs. First-Generation Cephalosporin + Metronidazole vs. Penicillin with Beta-Lactamase Inhibitor
Kalkanli (2018) (32)	2015-2017	Prospective cohort study	Urological surgery	Turkey	To compare the infectious complications of transrectal ultrasonography-guided prostate biopsy with and without extended antibiotic prophylaxis	antimicrobial resistance, infection	Fluoroquinolone vs. Fluoroquinolone
McCullough (2016) (34)	2005-2011	Retrospective cohort study	Plastic surgery	USA	To examine the incidence and bacteriology of SSI relative to patterns of postoperative antibiotic prophylaxis after expander-based breast reconstruction	antimicrobial resistance, infection (SSI)	First-Generation Cephalosporin + First-Generation Cephalosporin vs. First-Generation Cephalosporin + First-Generation Cephalosporin

Bartella (2017) (39)	2015-2016	Prospective cohort study	Head and neck surgery in patients with squamous cell carcinoma	Germany	To clarify the value of postoperative prophylactic antibiosis for the recovery and clinical course of patients.	antimicrobial resistance, infection	Penicillin with Beta-Lactamase Inhibitor vs. Penicillin with Beta-Lactamase Inhibitor vs. Penicillin with Beta-Lactamase Inhibitor
Rubinstein (1994) (44)	1986-1988	RCT	Spinal surgery	Israel	To evaluate the efficacy of a single preoperative dose of cephazolin in lumbar spinal surgery	antimicrobial resistance, infection (BSI, UTI)	First-Generation Cephalosporin vs. no prophylaxis
Yang (2013) (50)	2006-2010	Retrospective cohort study	Head and Neck Reconstructive Surgery	Taiwan	To identify bacterial components in saliva and its correlation to postsurgical infections7	antimicrobial resistance, infection (SSI)	Clindamycin + Gentamicin vs. no prophylaxis
Wilson (2008) (52)	-	secondary data from another study/implementation	Abdominal surgery	USA	To determine the potential cost impact of using ertapenem as opposed to cefotetan as prophylaxis for elective colorectal surgery	antimicrobial resistance, infection, LOS	Carbapenem vs. Second-Generation Cephalosporin
Gentilotti (2020) (53)	2013-2015	Prospective before after study	Gynecological surgery	Tanzania	To assess the impact of the implementation of a combined Infection Prevention and Control with Antimicrobial Stewardship joint program on the prevalence of caesarean section associated SSIs	antimicrobial resistance, infection (SSI)	Penicillin vs. Third-Generation Cephalosporin + Metronidazole

Amelot (2021) (66)	2011-2017	Retrospective before after study	Trauma surgery	France	To evaluate the role of preoperative antibiotic prophylaxis, to describe postoperative infections, their risk factors, and their bacteriology and to investigate the impact of antibiotic prophylaxis on the selection of resistant bacteria	antimicrobial resistance, infection (SSI), mortality	First-Generation Cephalosporin vs. no prophylaxis
Higaki (2022) (67)	2016-2020	Retrospective before after study	Esophagectomy	Japan	To establish which treatment, CEZ or ABPC/SBT, was better for the prevention of postoperative pneumonia	antimicrobial resistance, infection, LOS	First-Generation Cephalosporin vs. Penicillin with Beta-Lactamase Inhibitor
Longtin (2021) (69)	-	secondary data from another study/implementation	Heart and thoracic surgery	Canada, Netherlands	To compare the microbiology and antimicrobial susceptibility between patients receiving short-term or long-term antibiotic prophylaxis	antimicrobial resistance, infection	First-Generation Cephalosporin vs. First-Generation Cephalosporin + Vancomycin + First-Generation Cephalosporin
Dubinsky-Pertzov (2019) (70)	2012-2017	Prospective cohort study	Abdominal surgery	Israel, Switzerland, Serbia	To determine whether patients who are carriers of ESBL-PE have a higher risk of developing SSI after colorectal surgery than noncarriers when the routine prophylaxis regimen is used	antimicrobial resistance, infection (SSI)	Cephalosporin + Metronidazole vs. Cephalosporin + Metronidazole
Nutman (2020) (72)	2012-2017	Prospective cohort study	Abdominal surgery	Switzerland, Serbia, Israel	To test whether ESBL-PE screening of patients scheduled for elective colorectal surgery and personalized prophylaxis with ertapenem for those who	antimicrobial resistance, infection (SSI)	Second-Generation Cephalosporin + Metronidazole vs. Carbapenem

					screen positive reduces SSI risk among ESBL-PE carriers.		
Mestrallet (2023) (78)	2019-2022	Prospective cohort study	Head and neck	Belgium	To describe the infectious complications and to identify risk factors most commonly associated with infections.	antimicrobial resistance, infection (SSI), LOS	Penicillin with Beta-Lactamase Inhibitor vs. Penicillin with Beta-Lactamase Inhibitor
Khawcharoenporn (2022) (79)	2016-2019	Prospective cohort study	Urological surgery	Thailand	To compare post-TURP UTI incidence between patients receiving appropriate and inappropriate antibiotic prophylaxis and to analyze inappropriate antibiotic prophxylaxis rates, uropathogen resistance profiles, and UTI risk factors.	antimicrobial resistance, infection (UTI)	Third-Generation Cephalosporin vs. Fluoroquinolone
Kuo (2022) (80)	2008-2017	Retrospective cohort study	Trauma surgery	Taiwan	To evaluate the rate of PJI and antibiotic-related complications with the administration of extended antibiotic prophylaxis compared to standard antibiotic prophylaxis following aseptic revision hip and knee arthroplasty.	antimicrobial resistance, infection, LOS	First-Generation Cephalosporin vs. First-Generation Cephalosporin
Newman (2022) (82)	2014-2021	Retrospective cohort study	Urological surgery	UK	To evaluate the effect of augmented vs. targeted prophylaxis on the incidence of prostate biopsy-related sepsis.	antimicrobial resistance, infection (BSI)	Fluoroquinolone + Gentamicin vs. targeted prophylaxis + Gentamicin

Sewick (2012) (83)	2008-2010	Retrospective cohort study	Trauma surgery	USA	To determine whether dual antibiotic prophylaxis reduces SSI rates compared to single prophylaxis and alters SSI microbiology.	antimicrobial resistance, infection (SSI)	First-Generation Cephalosporin + Vancomycin vs. First-Generation Cephalosporin
Bains (2024) (84)	2014-2021	Retrospective cohort study	Trauma surgery	USA	To examine complications in primary total joint arthroplasty patients receiving vancomycin plus cefazolin versus cefazolin alone, focusing on infection microbiology, 30-day emergency department visits and readmissions, and infection risk factors.	antimicrobial resistance, infection (SSI)	First-Generation Cephalosporin + Vancomycin vs. First-Generation Cephalosporin
Bartella (2018) (85)	2016-2017	Prospective cohort study	Plastic surgery	Germany	To compare strict perioperative antibiotic treatment with extended prophylaxis until day 5 regarding SSI rates, hospitalization duration, and antibiotic-associated side effects.	antimicrobial resistance, infection (SSI)	Penicillin with Beta-Lactamase Inhibitor vs. Penicillin with Beta-Lactamase Inhibitor
Lopez (2019) (86)	2005-2015	Retrospective cohort study	Trauma surgery	USA	To assess whether administration of prophylactic vancomycin, in addition to cefazolin decreased revision surgeries for postoperative infection (SSI) as well as the need for revisions overall.	antimicrobial resistance, infection (SSI)	First-Generation Cephalosporin vs. First-Generation Cephalosporin + Vancomycin
Finkelstein (2002) (87)	1997-1999	RCT	Heart and thoracic surgery	Israel	To compare the efficacy of vancomycin prophylaxis with that of cefazolin in preventing surgical site infections in a tertiary medical center with a	antimicrobial resistance, infection (SSI), mortality, LOS	Vancomycin vs. First-Generation Cephalosporin

					high prevalence of methicillin-resistant staphylococcal infections.		
Ishibashi (2009)(88)	2003-2007	RCT	Abdominal surgery	Japan	To assess the effectiveness of short-term intravenous antimicrobial prophylaxis in combination with preoperative oral antibiotics on a surgical site and methicillin-resistant Staphylococcus aureus (MRSA) infection in elective colon cancer surgery.	antimicrobial resistance, infection (SSI)	Second-Generation Cephalosporin vs. Second-Generation Cephalosporin
Ishida (2001) (89)	1998-2000	RCT	Abdominal surgery	Japan	To evaluate the effect of administering preoperative oral antibiotics on the incidence of surgical site infection and MRSA infection in patients undergoing elective colorectal surgery.	antimicrobial resistance, infection (SSI)	Second-Generation Cephalosporin vs. Second-Generation Cephalosporin + other oral antibiotics
Kanayama (2007) (90)	1999-2004	Retrospective cohort study	Trauma surgery	Japan	To compare SSI rates in lumbar spine surgery between extended (5–7 days) and CDC-guideline-based single-day antimicrobial prophylaxis.	antimicrobial resistance, infection (SSI)	First-Generation Cephalosporin vs. First-Generation Cephalosporin
Kusachi (2008) (91)	1990-2006	Prospective cohort study	Abdominal surgery	Japan	To determine whether MRSA prevention is more effective with infection control agents given within 72 hours or 24 hours post-surgery.	antimicrobial resistance, infection (SSI)	Cephalosporin vs. Cephalosporin vs. Cephalosporin

Liu (2014) (92)	2008-2012	Retrospective before after study	Trauma surgery	USA	To assess the impact of targeted vancomycin use on periprosthetic joint infection incidence and methicillin-resistant prosthetic joint infections in revision total knee arthroplasty patients.	antimicrobial resistance, infection	First-Generation Cephalosporin vs. First-Generation Cephalosporin + Vancomycin
Mathur (2013) (93)	2009-2010	RCT	Trauma surgery	India	To evaluate the efficacy of a 24-hour perioperative antibiotic regimen in preventing SSIs after open reduction and internal fixation of closed limb fractures and its feasibility as a standard policy in a Level 1 Trauma Centre in India.	antimicrobial resistance, infection (SSI), LOS	Second-Generation Cephalosporin vs. Second-Generation Cephalosporin
Marigi (2024) (94)	2000-2019	Retrospective cohort study	Trauma surgery	USA	To evaluate the comparative efficacy of cefazolin and complete vancomycin administration for surgical prophylaxis in primary shoulder arthroplasty with respect to infectious complications.	antimicrobial resistance, infection, LOS	First-Generation Cephalosporin vs. Vancomycin
Peel (2023) (95)	2019-2022	RCT	Trauma surgery	Australia	To assess the efficacy of adding vancomycin to standard surgical antimicrobial prophylaxis with cefazolin to prevent surgical-site infection in adults undergoing arthroplasty.	antimicrobial resistance, infection (SSI), mortality	First-Generation Cephalosporin + Vancomycin vs. First-Generation Cephalosporin
Stallard (2022) (96)	2002-2005	RCT	Gynecological surgery	UK	To determine whether a single dose of amoxicillin-clavulanic acid reduces 30-day wound infections after primary breast	antimicrobial resistance, infection (SSI)	Penicillin with Beta-Lactamase Inhibitor vs. no prophylaxis

					cancer surgery and to identify infection risk factors.		
Nguyen (2019) (97)	2013-2016	Retrospective cohort study	Neurosurgery	USA	To determine if patients who underwent vancomycin prophylaxis instead of cefazolin prophylaxis experienced a significantly different incidence of SSIs following general neurosurgical procedures.	antimicrobial resistance, infection (SSI)	First-Generation Cephalosporin vs. Vancomycin
Cao (2017) (98)	2009-2015	Retrospective cohort study	Neurosurgery	China	To investigate the efficacy of antibiotic prophylaxis (AP) and the risk factors for postoperative infections in clean neurosurgery.	antimicrobial resistance, infection	Second-Generation Cephalosporin vs. no prophylaxis
Manecksha (2012) (99)	2008-2009	Prospective cohort study	Urological surgery	Ireland	To compare infection rates of two 3-day antibiotic prophylaxis regimens for TRUS-guided prostate biopsy and analyze local microbiological trends.	antimicrobial resistance, infection (UTI, BSI)	Fluoroquinolone vs. Fluoroquinolone
Patrick (2010) (100)	2007-2009	RCT	Heart and thoracic surgery	USA	To evaluate whether adding anti-MRSA antibiotics to standard prophylaxis reduces overall and MRSA-related infections in low-risk patients through a prospective randomized trial comparing cefazolin alone vs. combination therapy.	antimicrobial resistance, infection (SSI)	First-Generation Cephalosporin vs. Vancomycin + First-Generation Cephalosporin vs. Daptomycin + First-Generation Cephalosporin

Fahmy (2016) (101)	2012-2015	RCT	Urological surgery	Egypt	To compare the incidence of infectious complications after single-dose fosfomycin vs. standard fluoroquinolone (FQ)-based prophylaxis in patients undergoing transrectal ultrasound-guided biopsy of the prostate (TRUSBx).	antimicrobial resistance, infection (UTI)	Fosfomycin vs. Fluoroquinolone + Metronidazole
Pace (2012) (102)	2010-2011	RCT	Urological surgery	Italy	To compare oral quinolone prophylaxis with a combination of periprostatic cephalosporin and oral fluoroquinolone regarding post-biopsy infection rates in men undergoing TRUS-guided prostate biopsy.	antimicrobial resistance, infection (UTI, BSI)	Fluoroquinolone vs. Third-Generation Cephalosporin + Fluoroquinolone
Ongun (2012) (103)	2010-2011	Retrospective cohort study	Urological surgery	Turkey	To evaluate the efficacy of single-dose fosfomycin prophylaxis as an alternative to fluoroquinolone-based prophylaxis in transrectal ultrasound-guided biopsy of the prostate (TRUSBP).	antimicrobial resistance, infection	Fluoroquinolone vs. Fluoroquinolone vs. Fosfomycin
Haga (2012) (104)	2007-2010	RCT	Abdominal surgery	Japan	To show that the single-dose prophylaxis was not inferior to the multiple-dose prophylaxis in terms of the overall incidence of SSIs.	antimicrobial resistance, infection (SSI)	First-Generation Cephalosporin vs. First-Generation Cephalosporin
Ho (2009) (105)	2003-2005	Mixed (Prosp. & Retrosp.) cohort study	Urological surgery	Singapore	To review post-TRPB sepsis cases and their bacteriology, introduce intramuscular gentamicin alongside oral ciprofloxacin, and compare	antimicrobial resistance, infection (BSI), LOS	Fluoroquinolone vs. Fluoroquinolone + Gentamicin

					sepsis incidence before and after this change.		
Lista (2014) (106)	2009-2010	RCT	Urological surgery	Spain	To evaluate the efficacy and safety of antibiotic prophylaxis for prostate biopsy by comparing two doses of fosfomycin-trometamol with a 5-day ciprofloxacin regimen.	antimicrobial resistance, infection (UTI)	Fluoroquinolone vs. Fosfomycin
Mohri (2007) (107)	2001-2004	RCT	Abdominal surgery	Japan	To compare single-dose versus multiple-dose antimicrobial prophylaxis for preventing surgical site infections in a multicenter randomized clinical trial.	antimicrobial resistance, infection (SSI)	Cephalosporin or Penicillin with Beta-Lactamase Inhibitor vs. Cephalosporin or Penicillin with Beta-Lactamase Inhibitor
De Pastena (2021) (108)	2015-2018	Prospective before after study	Abdominal surgery	Italy	To evaluate the effectiveness of piperacillin-tazobactam as antibiotic prophylaxis in patients affected by a peri-ampullary tumor submitted to pancreatic surgery.	antimicrobial resistance, infection (SSI)	Penicillin with Beta-Lactamase Inhibitor vs. Pip_BLI
Tang (2018) (109)	2012-2017	Retrospective cohort study	Abdominal surgery	China	To evaluate the efficacy of a single dose of ertapenem within 30 minutes to two hours prior to skin incision in preventing SSI in patients with hepatocellular carcinoma (HCC).	antimicrobial resistance, infection (SSI)	Carbapenem vs. non-Carbapenem prophylaxis

Supplementary Figure S1: Forest plot of the relative risk of resistance rates against prophylactic agent among bacterial infections in hemato-oncological patients receiving prophylaxis as compared to those not receiving prophylaxis reported between 1991-2024, classified by country of study (N=21)

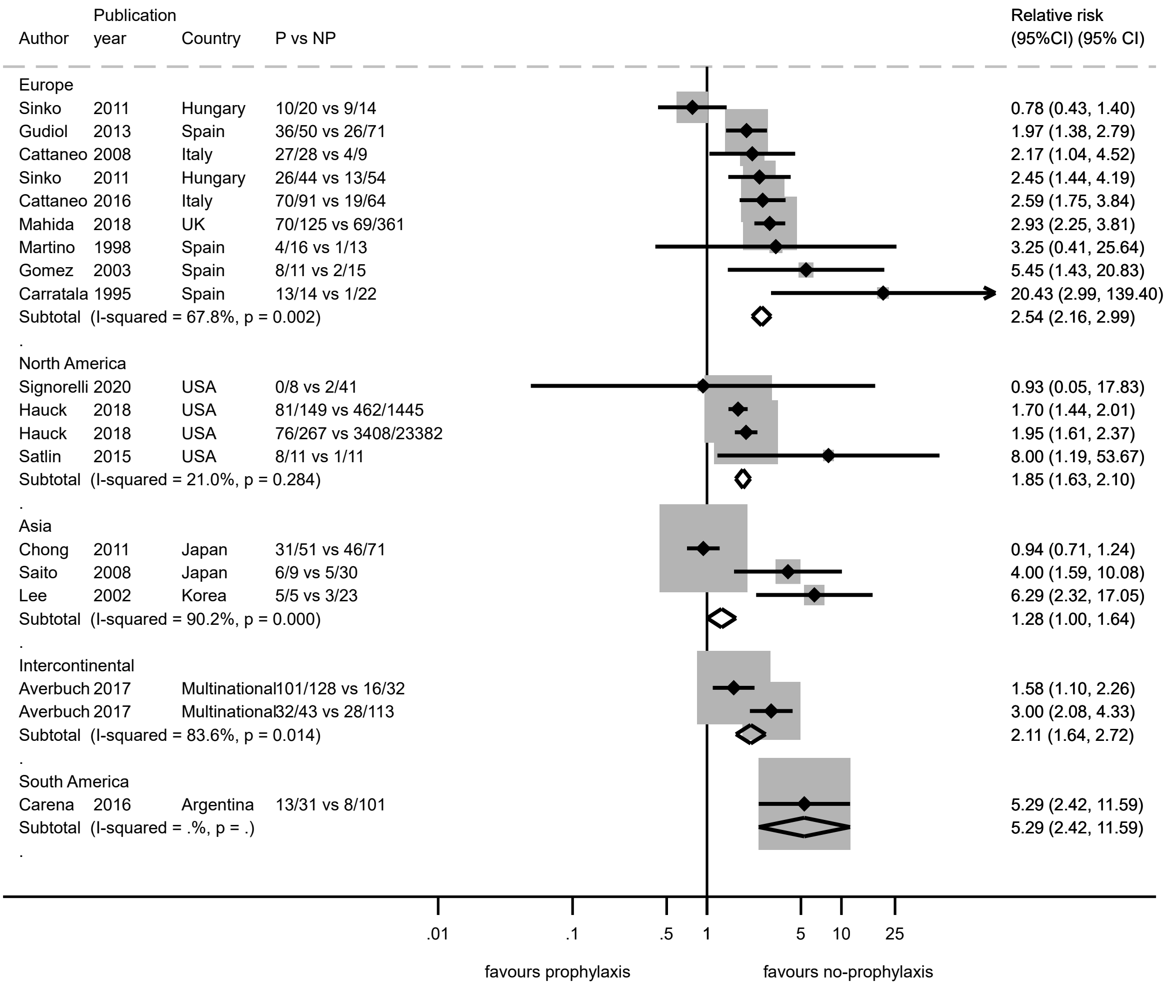

Supplementary Figure S2: Forest plot of the relative risk of resistance rates against prophylactic agent among bacterial infections in hemato-oncological patients receiving prophylaxis as compared to those not receiving prophylaxis reported between 1991-2024, classified by year of publication (N=21)

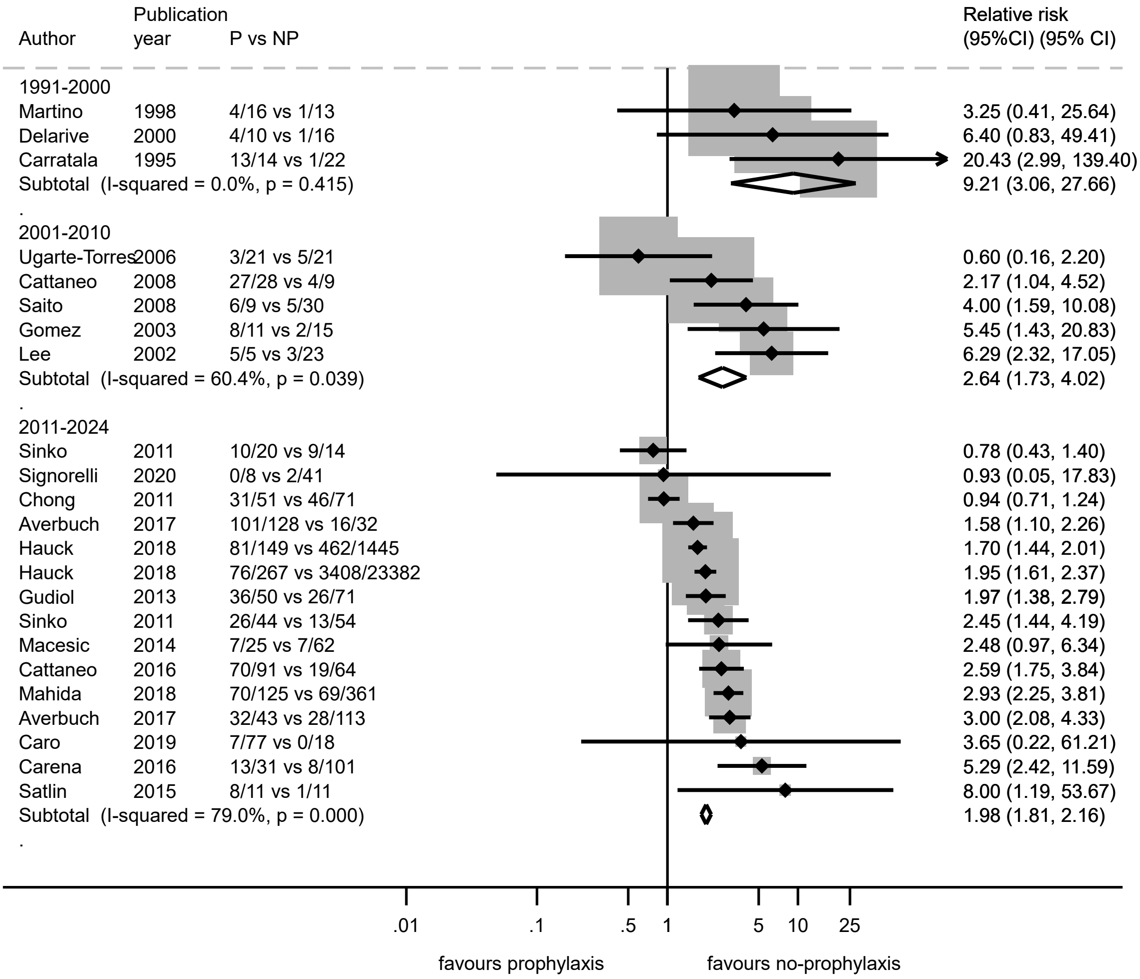

Supplementary Table S2: Pooled estimates of resistance rates among causative pathogens with any antibiotic resistance in comparison with patients not receiving prophylaxis in hemato-oncological studies among subgroups of interest with three or more contributing studies each (N=21)

**Subgroup**	**Category**	**N_s (N_o)***	**Risk Ratio (95% CI)**	**p-value**	**I-square**	**p-value for I-square**
Causative pathogen studied	All bacteria	5 (8)	1.64 (1.2 - 2.23)	0.002	11.5%	0.340
	Enterobacterales	4 (6)	2.73 (2.25 - 3.32)	<0.001	0.0%	0.602
	Escherichia coli	9 (14)	1.78 (1.58 - 1.99)	<0.001	58.9%	0.003
	Gram negative bacteria	8 (16)	2.07 (1.76 - 2.45)	<0.001	75.3%	<0.001
	Gram positive bacteria	3 (4)	0.88 (0.69 - 1.12)	0.313	50.4%	0.109
Pathogen grouped	grouped Gram positive bacteria	7 (8)	1.12 (0.89 - 1.41)	0.340	64.8%	0.006
	grouped Gram negative bacteria	18 (38)	2.03 (1.86 - 2.21)	<0.001	68%	<0.001
Combination of prophlyactic agent and resistance	Quinolone prophylaxis and Carbapenem resistance	4 (5)	0.92 (0.59 - 1.43)	0.715	46.3%	0.114
	Quinolone prophylaxis and ESBL	3 (3)	1.82 (1.04 - 3.18)	0.037	0.0%	0.436
	Quinolone prophylaxis and MDR	4 (5)	1.74 (1.32 - 2.30)	<0.001	87%	<0.001
	Quinolone prophylaxis and Quinolone resistance	17 (21)	2 (1.83 - 2.19)	<0.001	73.5%	<0.001
Continents	Asia	4 (6)	1.29 (1.02 - 1.62)	0.035	76.7%	0.001
	Europe	9 (15)	2.17 (1.86 - 2.54)	<0.001	76.9%	<0.001
	North America	4 (8)	1.81 (1.6 - 2.05)	<0.001	4.7%	0.394
Stemcell transplantation	no	3 (4)	2.01 (1.43 - 2.82)	<0.001	61.9%	0.048
	partly	8 (14)	1.72 (1.54 - 1.92)	<0.001	69.7%	<0.001
	unknown	3 (3)	1.5 (1.14 - 1.96)	0.003	90.7%	<0.001
	yes	7 (19)	2.24 (1.94 - 2.58)	<0.001	68%	<0.001
Publication year	1991-2000	3 (5)	1.96 (1.21 - 3.2)	0.007	84.7%	<0.001
	2001-2010	5 (6)	2.33 (1.61 - 3.37)	<0.001	73.7%	0.002
	2011-2024	13 (29)	1.87 (1.72 - 2.04)	<0.001	72.0%	<0.001

*N_s (N_o) refers to number of studies (number of outcomes)

Supplementary Figure S3: Forest plot of the weighted mean difference of length of hospital stay between hemato-oncological patients receiving prophylaxis as compared to those not receiving prophylaxis reported between 1991-2024 (N=5)

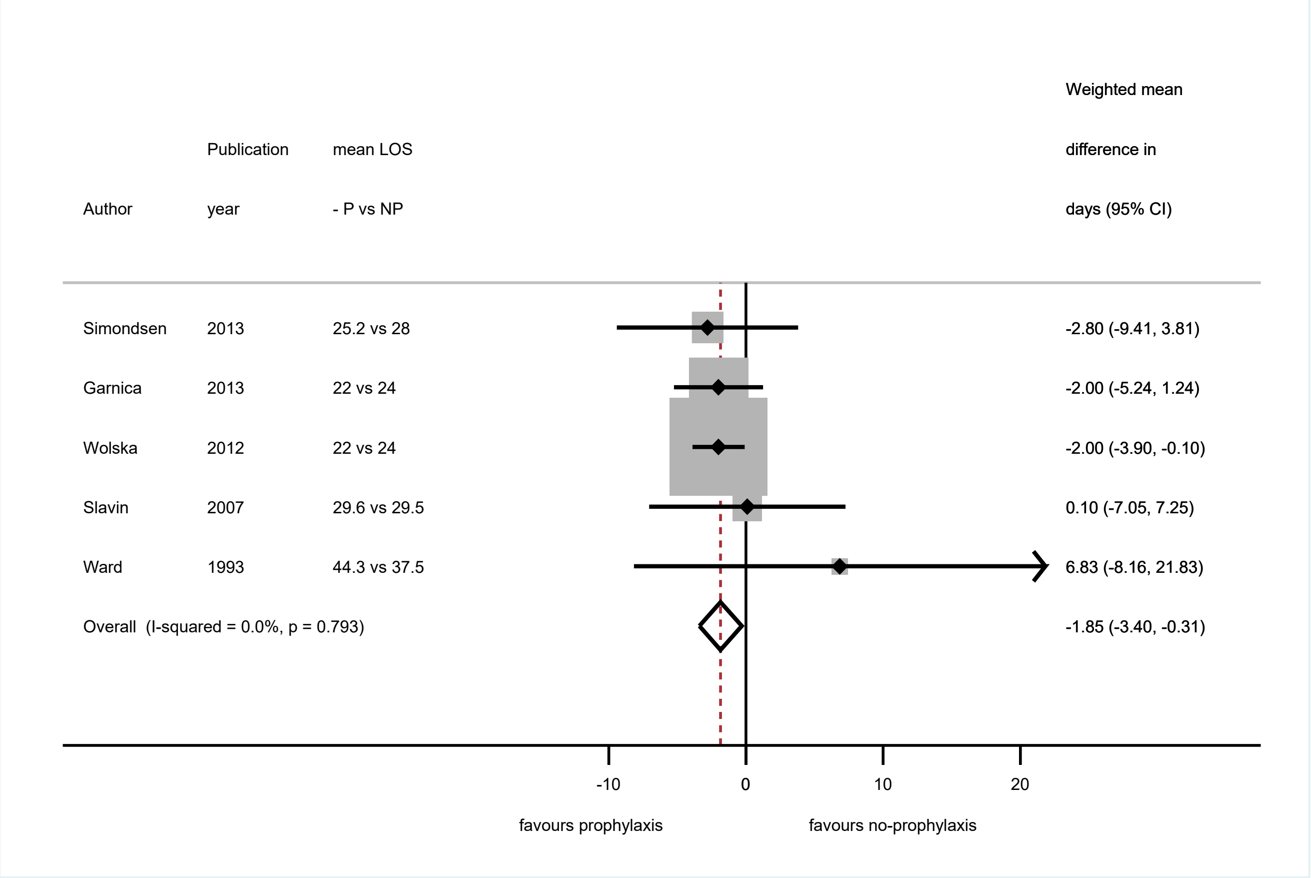

Supplementary Figure S4: Forest plot of the relative risk of surgical site infections between surgical patients receiving prophylaxis as compared to those not receiving prophylaxis reported between 1991-2024 (N=5)

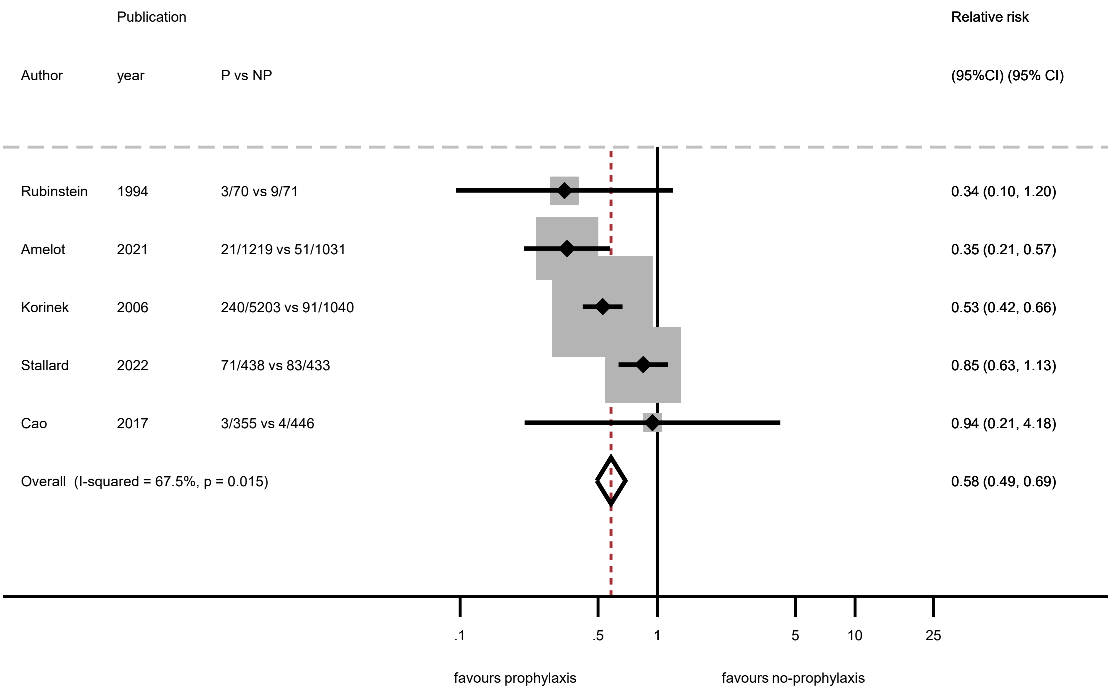

Supplementary Figure S5: Forest plot of the relative risk of any infection among surgical patients receiving short prophylaxis as compared to those on extended prophylaxis reported between 1991-2024 (N=14)

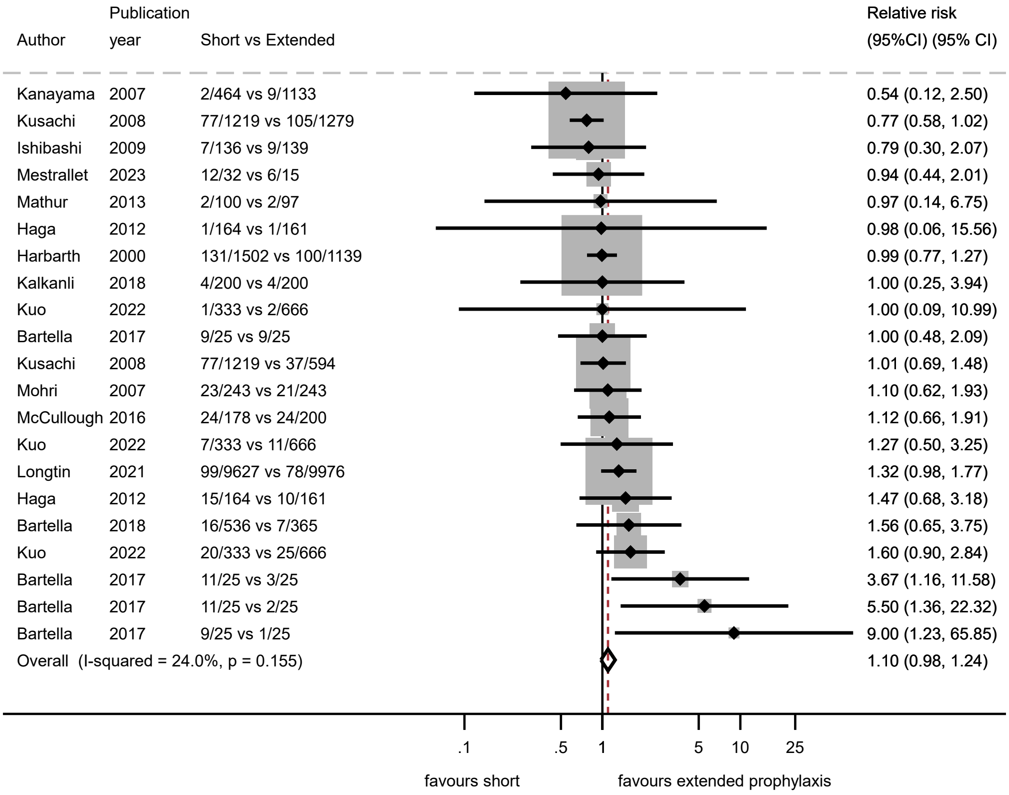

Supplementary Figure S6: Risk of bias assessment of the included hemato-oncological studies with a comparison group using modified Joanna Briggs Institute's critical appraisal tool (N=55)

Risk of bias

Study	D1	D2	D3	D4	D5	D6	D7	D8	D9	D10	D11	Overall
Akhmedov 2023	+	+	+	+	+	+	+	+	+	+	+	+
Averbuch 2017	-	+	+	+	-	+	+	+	+	+	+	-
Carena 2016	+	+	+	+	+	+	+	+	+	+	X	-
Caro 2019	X	+	+	+	+	+	+	+	+	+	+	-
Caro 2022	+	+	+	+	+	+	+	+	+	X	+	-
Carratala 1995	-	+	+	+	-	+	+	X	+	X	X	X
Cattaneo 2008	-	+	+	+	+	+	+	+	+	+	+	+
Cattaneo 2016	-	+	+	+	-	+	+	+	+	+	+	-
Chong 2011	-	+	+	+	+	+	+	+	+	X	X	X
Clerici 2023	+	+	+	+	+	+	+	+	+	+	+	+
Craig M 2007	-	+	+	+	+	+	+	+	+	X	X	X
Cumpston 2013	-	+	+	+	+	+	+	+	+	X	X	X
D'Antonio 1994	+	+	+	+	+	+	+	+	+	+	+	+
Delarive 2000	+	+	+	+	+	+	+	+	+	X	X	-
Engels 1999	-	+	+	+	-	+	+	+	+	+	+	-
Ganti 2017	+	+	+	+	+	+	+	+	+	X	X	-
Garnica 2013	X	+	+	+	+	+	+	+	+	X	X	X
Gomez 2003	+	+	+	+	+	+	+	+	+	X	X	-
Guare 2024	+	+	+	+	+	+	+	+	+	+	+	+
Gudiol 2013	X	+	+	+	+	+	+	+	+	X	X	X
Guimaraes 2022	+	+	+	+	+	+	+	+	+	+	+	+
Guiot 1992	+	+	+	+	+	+	+	+	+	+	+	+
Hakki 2019	X	+	+	+	+	+	+	+	+	X	X	X
Hauck 2018	-	+	+	+	+	+	+	+	+	+	+	+
Hsueh 2005	X	+	+	+	+	+	+	+	X	X	X	X
Kern 2005	-	+	+	+	+	+	+	+	+	X	X	X
Kern 2018	-	+	+	+	+	+	+	+	+	+	+	+
Lee 2002	+	+	+	+	+	+	+	+	+	+	+	+
Lee 2017	-	+	+	+	+	+	+	X	+	X	X	X

Macesic 2014	+	+	+	+	+	+	+	+	X	-	-	-
Mahida 2018	-	+	+	+	+	+	+	+	+	X	X	X
Martino 1998	+	+	+	+	+	+	+	+	+	X	X	-
Munoz 1999	X	+	+	+	+	+	+	+	+	+	X	-
Nucci 1994	+	+	+	+	+	+	+	+	+	X	X	-
Prentice 2001	+	+	+	+	+	+	+	+	X	X	X	-
Qin 2007	X	+	+	+	+	+	+	+	+	X	X	X
Saito 2008	+	+	+	+	+	+	+	+	+	+	X	-
Satlin 2015	+	+	+	+	+	+	+	+	+	X	X	-
Satlin 2021	+	+	+	+	+	+	+	+	+	+	+	+
Schroeder 1992	+	+	+	+	+	+	+	+	+	X	X	-
Signorelli 2020	-	+	+	+	+	+	+	+	+	+	+	+
Simondsen 2013	-	+	+	+	+	+	+	+	+	X	X	X
Sinko 2011	-	+	+	+	+	+	+	+	+	X	X	X
Slavin 2007	+	+	+	+	+	+	+	+	+	+	+	+
Sohn 2012	X	+	+	+	+	+	+	+	+	X	X	X
Sojo 2016	X	+	+	+	+	+	+	+	+	X	X	X
Timmers 2007	+	+	+	+	+	+	+	+	+	+	+	+
Trecarichi 2019	+	+	+	+	+	+	+	+	+	+	+	+
Ugarte-Torres 2006	X	+	+	+	+	+	+	+	+	+	X	-
Urbino 2023	+	+	+	+	+	+	+	+	+	X	+	-
Verlinden 2014	+	+	+	+	+	+	+	+	+	X	X	-
Ward 1993	+	+	+	+	+	+	+	+	+	+	X	-
Wolska 2012	+	+	+	+	+	+	+	+	+	X	X	-
Zaidi 2001	X	+	X	+	-	+	+	+	X	X	X	X
Zavrelova 2019	-	+	+	+	+	+	+	+	+	X	X	X

D1: Similar comparison groups?
D2: Temporal causality?
D3: Similar assessment of exposures?
D4: Valid assessment of exposure?
D5: Similar evaluation of outcomes?
D6: Valid measurement of outcome?
D7: Sufficient follow-up time for outcome?
D8: Complete follow-up?
D9: Comparison
D10: Confounding
D11: Appropriate statistical analysis used?

Judgement
X High
- Moderate
+ Low

Supplementary Figure S7: Summary of the risk of bias assessment of the included hemato-oncological studies with a comparison group using modified Joanna Briggs Institute's critical appraisal tool (N=55)

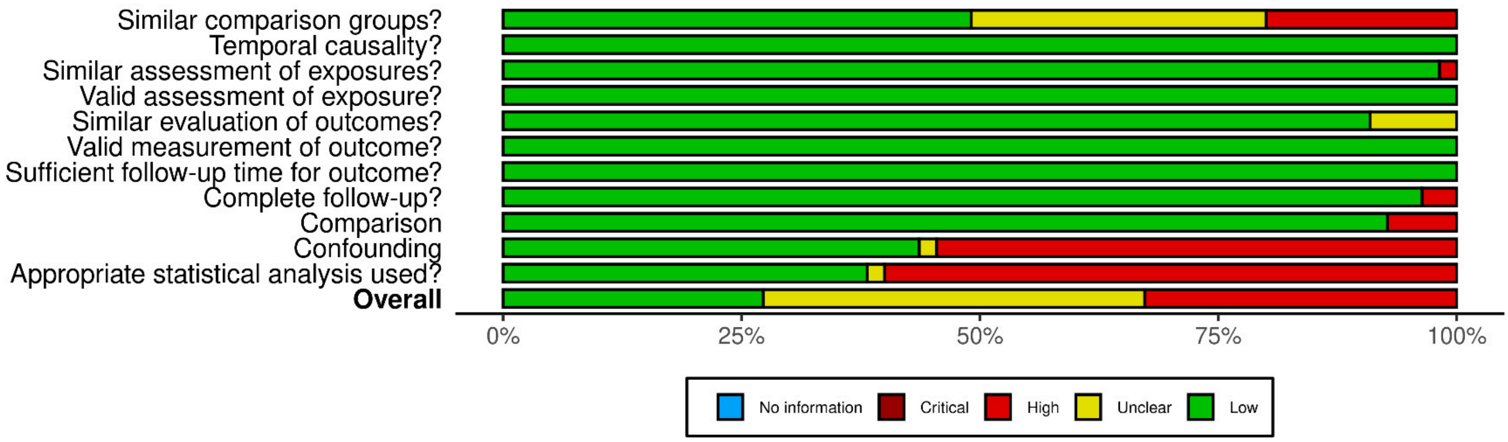

Supplementary Figure S8: Risk of bias assessment of the included surgical studies with a comparison group using modified Joanna Briggs Institute's critical appraisal tool (N=54)

Risk of bias

Study	D1	D2	D3	D4	D5	D6	D7	D8	D9	D10	D11	vera
Adibi 2013	+	+	+	+	+	+	+	+	+	X	X	-
Amelot 2021	+	+	+	+	+	+	+	+	+	+	+	+
Antsupova 2014	+	+	+	+	+	+	+	+	+	X	X	-
Bains 2024	+	+	+	+	+	+	+	+	+	X	+	-
Bartella 2017	+	+	+	+	+	+	+	+	+	X	X	-
Bartella 2018	+	+	+	+	+	+	+	+	+	X	+	-
Cammann 2016	+	+	+	+	+	+	+	+	+	X	X	-
Cao 2017	+	+	+	+	+	+	+	+	+	+	+	+
Cohen 2017	-	+	+	+	+	+	+	+	+	+	+	+
De Pastena 2021	+	+	+	+	+	+	+	+	+	X	+	-
Dubinsky-Pertzov 2019	-	+	+	+	+	+	+	+	+	+	+	+
Fahmy 2016	+	+	+	+	+	+	+	+	+	X	X	-
Finkelstein 2002	+	+	+	+	+	+	+	+	+	X	X	-
Fong 2016	+	+	+	+	+	+	+	+	+	+	+	+
Gentilotti 2020	X	+	+	+	+	+	+	+	+	X	X	X
Goldstein 2009	+	+	+	+	+	+	+	+	+	+	+	+
Haga 2012	+	+	+	+	+	+	+	+	+	+	+	+
Harbarth 2000	+	+	+	+	+	+	+	+	+	+	+	+
Higaki 2022	+	+	+	+	+	+	+	+	+	+	+	+
Ho 2009	+	+	+	+	+	+	+	+	+	X	X	-
Ishibashi 2009	+	+	+	+	+	+	+	+	+	+	+	+
Ishida 2001	+	+	+	+	+	+	+	+	+	X	+	-
Itani 2006	+	+	+	+	+	+	+	+	+	+	+	+
Kalkanli 2018	+	+	+	+	+	+	+	+	+	X	X	-
Kanayama 2007	+	+	+	+	+	+	+	+	+	X	X	-
Khawcharoenporn 2022	+	+	+	+	+	+	+	+	+	X	+	-
Korinek 2006	-	+	+	+	+	+	+	+	+	X	X	X
Kuo 2022	+	+	+	+	+	+	+	+	+	+	+	+

Kusachi 2008	+	+	+	+	+	+	+	+	+	X	X	-
Lee 2016	+	+	+	+	+	+	+	+	+	+	+	+
Lista 2014	+	+	+	+	+	+	+	+	+	X	X	-
Liu 2014	+	+	+	+	+	+	+	+	+	X	X	-
Longtin 2021	+	+	+	+	+	+	+	+	+	+	+	+
Lopez 2019	+	+	+	+	+	+	+	+	+	+	X	-
Maneckshа 2012	+	+	+	+	+	+	+	+	+	X	X	-
Marigi 2024	+	+	+	+	+	+	+	+	+	+	+	+
Mathur 2013	+	+	+	+	+	+	+	+	+	X	X	-
McCullough 2016	+	+	+	+	+	+	+	+	+	X	X	-
Merrer 2006	-	+	+	+	+	+	+	+	+	X	X	X
Mestrallet 2023	+	+	+	+	+	+	+	+	+	X	X	-
Mohri 2007	+	+	+	+	+	+	+	+	+	X	X	-
Newman 2022	+	+	+	+	+	+	+	+	+	X	X	-
Nguyen 2019	+	+	+	+	+	+	+	+	+	+	+	+
Nutman 2020	+	+	+	+	+	+	+	+	+	+	+	+
Ongün 2012	+	+	+	+	+	+	+	+	+	X	X	-
Pace 2012	+	+	+	+	+	+	+	+	+	X	X	-
Patrick 2010	+	+	+	+	+	+	+	+	+	X	+	-
Peel 2023	+	+	+	+	+	+	+	+	+	+	X	-
Rubinstein 1994	+	+	+	+	+	+	+	+	+	X	X	-
Sewick 2012	+	+	+	+	+	+	+	+	+	+	+	+
Stallard 2022	+	+	+	+	+	+	+	+	+	X	X	-
Tang 2018	+	+	+	+	+	+	+	+	+	X	X	-
Wilson 2008	+	+	+	+	+	+	+	+	+	+	+	+
Yang 2013	+	+	+	+	+	+	+	+	+	X	X	-

D1: Similar comparison groups?
D2: Temporal causality?
D3: Similar assessment of exposures?
D4: Valid assessment of exposure?
D5: Similar evaluation of outcomes?
D6: Valid measurement of outcome?
D7: Sufficient follow-up time for outcome?
D8: Complete follow-up?
D9: Was there a control group?
D10: Confounding
D11: Appropriate statistical analysis used?

Judgement
X High
- Moderate
+ Low

Supplementary Figure S9: Summary of the risk of bias assessment of the included surgical studies with a comparison group using modified Joanna Briggs Institute's critical appraisal tool (N=54)

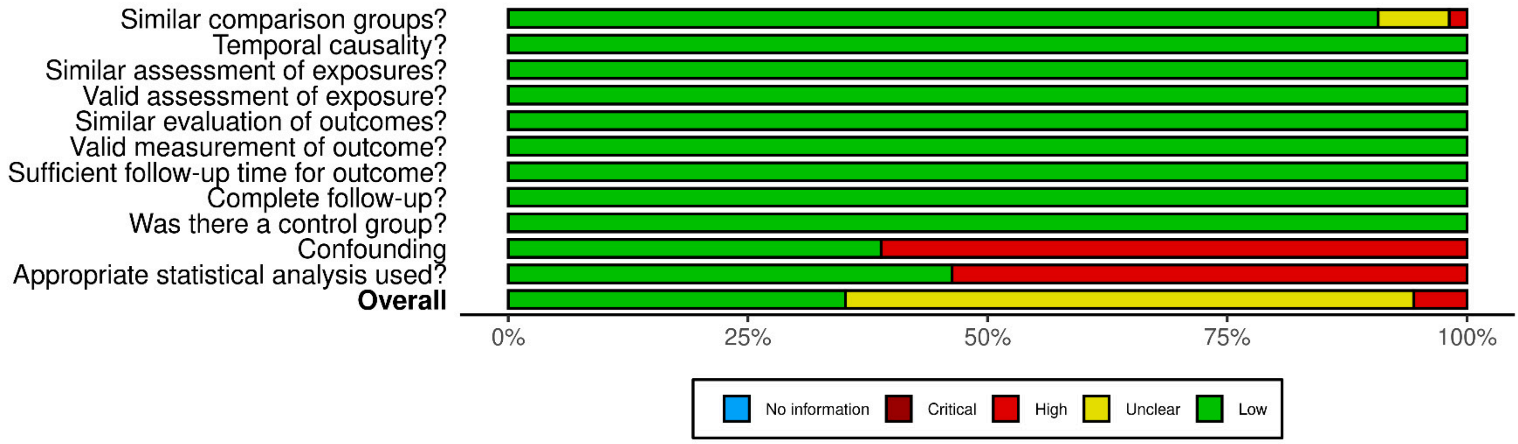

References

1. Antsupova V, Norgaard N, Bisbjerg R, Jensen JN, Boel J, Jarlov JO, et al. Antibiotic prophylaxis for transrectal prostate biopsy-a new strategy. Journal of Antimicrobial Chemotherapy. 2014;69(12):3372-8.
2. Signorelli J, Zimmer A, Liewer S, Shostrom VK, Freifeld A. Incidence of Febrile Neutropenia in Autologous Hematopoietic Stem Cell Transplant (HSCT) Recipients on levofloxacin prophylaxis. Transplant Infectious Disease. 2020;22(2).
3. Craig M, Cumpston AD, Hobbs GR, DeVetten MP, Sarwari AR, Ericson SG. The clinical impact of antibacterial prophylaxis and cycling antibiotics for febrile neutropenia in a hematological malignancy and transplantation unit. Bone Marrow Transplantation. 2007;39(8):477-82.
4. Zaidi Y, Hastings M, Murray J, Hassan R, Kurshid M, Mahendra P. Quinolone resistance in neutropenic patients: the effect of prescribing policy in the UK and Pakistan. Clinical and Laboratory Haematology. 2001;23(1):39-42.
5. Gomez L, Garau J, Estrada C, Marquez M, Dalmau D, Xercavins M, et al. Ciprofloxacin prophylaxis in patients with acute leukemia and granulocytopenia in an area with a high prevalence of ciprofloxacin-resistant Escherichial coli. Cancer. 2003;97(2):419-24.
6. Timmers GJ, Simoons-Smit AM, Leidekker ME, Janssen J, Vandenbroucke-Grauls C, Huijgens PC. Levofloxacin vs. ciprofloxacin plus phenethicillin for the prevention of bacterial infections in patients with haematological malignancies. Clinical Microbiology and Infection. 2007;13(5):497-503.
7. Chong Y, Yakushiji H, Ito Y, Kamimura T. Clinical impact of fluoroquinolone prophylaxis in neutropenic patients with hematological malignancies. International Journal of Infectious Diseases. 2011;15(4):E277-E81.
8. Wolska A, Robak T, Szmigielska-Kaplon A, Pluta A, Kopka P, Wierzbowska A. Ciprofloxacin prophylaxis for patients undergoing high-dose chemotherapy and autologous stem cell transplantation (ASCT) - a single-center experience. Advances in Medical Sciences. 2012;57(1):118-23.
9. Cattaneo C, Quaresmini G, Casari S, Capucci MA, Micheletti M, Borlenghi E, et al. Recent changes in bacterial epidemiology and the emergence of fluoroquinolone-resistant Escherichia coli among patients with haematological malignancies: results of a prospective study on 823 patients at a single institution. Journal of Antimicrobial Chemotherapy. 2008;61(3):721-8.
10. Carratalá J, Fernández-Sevilla A, Tubau F, Callis M, Gudiol F. Emergence of quinolone-resistant Escherichia coli bacteremia in neutropenic patients with cancer who have received prophylactic norfloxacin. Clin Infect Dis. 1995;20(3):557-60; discussion 61-3.
11. Hakki M, Humphries RM, Hemarajata P, Tallman GB, Shields RK, Mettus RT, et al. Fluoroquinolone Prophylaxis Selects for Meropenem-nonsusceptible Pseudomonas aeruginosa in Patients With Hematologic Malignancies and Hematopoietic Cell Transplant Recipients. Clinical Infectious Diseases. 2019;68(12):2045-52.
12. Korinek AM, Baugnon T, Golmard JL, van Effenterre R, Coriat P, Puybasset L. Risk factors for adult nosocomial meningitis after craniotomy: Role of antibiotic prophylaxis. Neurosurgery. 2006;59(1):126-32.
13. Lee SSF, Fulford AE, Quinn MA, Seabrook J, Rajakumar I. Levofloxacin for febrile neutropenia prophylaxis in acute myeloid leukemia patients associated with reduction in hospital admissions. Supportive Care in Cancer. 2018;26(5):1499-504.
14. Kern WV, Weber S, Dettenkofer M, Kaier K, Bertz H, Behnke M, et al. Impact of fluoroquinolone prophylaxis during neutropenia on bloodstream infection: Data from a surveillance program in 8755 patients receiving high-dose chemotherapy for haematologic malignancies between 2009 and 2014. J Infect. 2018;77(1):68-74.
15. Goldstein EJ, Citron DM, Merriam CV, Abramson MA. Infection after elective colorectal surgery: bacteriological analysis of failures in a randomized trial of cefotetan vs. ertapenem prophylaxis. Surg Infect (Larchmt). 2009;10(2):111-8.

16. Lee JK, Lee S, Hong SK, Byun SS, Lee SE. Clinical importance of the antibiotic regimen in transrectal ultrasound-guided biopsy: quinolone versus cephalosporin. Bmc Urology. 2016;16.
17. Hauck CG, Chong PP, Miller MB, Jamieson K, Fine JP, Foster MC, et al. Increasing Rates of Fluoroquinolone Resistance in Escherichia coli Isolated From the Blood and Urine of Patients with Hematologic Malignancies and Stem Cell Transplant Recipients. Pathog Immun. 2016;1(2):234-42.
18. Itani KM, Wilson SE, Awad SS, Jensen EH, Finn TS, Abramson MA. Ertapenem versus cefotetan prophylaxis in elective colorectal surgery. N Engl J Med. 2006;355(25):2640-51.
19. Engels EA, Ellis CA, Supran SE, Schmid CH, Barza M, Schenkein DP, et al. Early infection in bone marrow transplantation: Quantitative study of clinical factors that affect risk. Clinical Infectious Diseases. 1999;28(2):256-66.
20. Gudiol C, Bodro M, Simonetti A, Tubau F, Gonzalez-Barca E, Cisnal M, et al. Changing aetiology, clinical features, antimicrobial resistance, and outcomes of bloodstream infection in neutropenic cancer patients. Clinical Microbiology and Infection. 2013;19(5):474-9.
21. Harbarth S, Samore MH, Lichtenberg D, Carmeli Y. Prolonged antibiotic prophylaxis after cardiovascular surgery and its effect on surgical site infections and antimicrobial resistance. Circulation. 2000;101(25):2916-21.
22. Hsueh PR, Cheng HJ, Tang JL, Yao M, Tien HF. Prophylactic use of moxifloxacin in patients receiving bone marrow transplants was not associated with increased ciprofloxacin resistance in Escherichia coli and enterococci. Clinical Infectious Diseases. 2005;40(12):1862-4.
23. Ward TT, Thomas RG, Fye CL, Arbeit R, Coltman CA, Craig W, et al. Trimethoprim-sulfamethoxazole prophylaxis in granulocytopenic patients with acute leukemia: evaluation of serum antibiotic levels in a randomized, double-blind, placebo-controlled Department of Veterans Affairs Cooperative Study. Clinical infectious diseases. 1993;17(3):323-32.
24. Cattaneo C, Zappasodi P, Mancini V, Annaloro C, Pavesi F, Skert C, et al. Emerging resistant bacteria strains in bloodstream infections of acute leukaemia patients: results of a prospective study by the Rete Ematologica Lombarda (Rel). Annals of Hematology. 2016;95(12):1955-63.
25. Adibi M, Hornberger B, Bhat D, Raj G, Roehrborn CG, Lotan Y. Reduction in Hospital Admission Rates Due to Post-Prostate Biopsy Infections After Augmenting Standard Antibiotic Prophylaxis. Journal of Urology. 2013;189(2):535-40.
26. Cohen ME, Salmasian H, Li J, Liu J, Zachariah P, Wright JD, et al. Surgical Antibiotic Prophylaxis and Risk for Postoperative Antibiotic-Resistant Infections. J Am Coll Surg. 2017;225(5):631-8.e3.
27. Merrer J, Desbouchages L, Serazin V, Razafimamonjy J, Pauthier F, Leneveu M. Comparison of routine prophylaxis with vancomycin or cefazolin for femoral neck fracture surgery: Microbiological and clinical outcomes. Infection Control and Hospital Epidemiology. 2006;27(12):1366-71.
28. Cammann S, Timrott K, Vonberg RP, Vondran FWR, Schrem H, Suerbaum S, et al. Cholangitis in the postoperative course after biliodigestive anastomosis. Langenbecks Archives of Surgery. 2016;401(5):715-24.
29. Cumpston A, Craig M, Hamadani M, Abraham J, Hobbs GR, Sarwari AR. Extended follow-up of an antibiotic cycling program for the management of febrile neutropenia in a hematologic malignancy and hematopoietic cell transplantation unit. Transplant Infectious Disease. 2013;15(2):142-9.
30. Fong ZV, McMillan MT, Marchegiani G, Sahora K, Malleo G, De Pastena M, et al. Discordance Between Perioperative Antibiotic Prophylaxis and Wound Infection Cultures in Patients Undergoing Pancreaticoduodenectomy. Jama Surgery. 2016;151(5):432-9.
31. Ganti BR, Marini BL, Nagel J, Bixby D, Perissinotti AJ. Impact of antibacterial prophylaxis during reinduction chemotherapy for relapse/refractory acute myeloid leukemia. Supportive Care in Cancer. 2017;25(2):541-7.
32. Kalkanli A, Gezmis CT, Ozkan A, Cilesiz NC, Yanaral F, Aydin M, et al. Comparison of Single and Prolonged Fluoroquinolone Prophylaxis and Risk Factors for Infectious Complications After Transrectal Prostate Biopsy. Balkan Medical Journal. 2018;35(5):373-7.
33. Kern WV, Klose K, Jellen-Ritter AS, Oethinger M, Bohnert J, Kern P, et al. Fluoroquinolone resistance of Escherichia coli at a cancer center: epidemiologic evolution and effects of discontinuing

prophylactic fluoroquinolone use in neutropenic patients with leukemia. European Journal of Clinical Microbiology & Infectious Diseases. 2005;24(2):111-8.
34. McCullough MC, Chu CK, Duggal CS, Losken A, Carlson GW. Antibiotic Prophylaxis and Resistance in Surgical Site Infection After Immediate Tissue Expander Reconstruction of the Breast. Annals of Plastic Surgery. 2016;77(5):501-5.
35. Prentice HG, Hann IM, Nazareth B, Paterson P, Bhamra A, Kibbler CC. Oral ciprofloxacin plus colistin: prophylaxis against bacterial infection in neutropenic patients. A strategy for the prevention of emergence of antimicrobial resistance. British journal of haematology. 2001;115(1):46-52.
36. Satlin MJ, Vardhana S, Soave R, Shore TB, Mark TM, Jacobs SE, et al. Impact of Prophylactic Levofloxacin on Rates of Bloodstream Infection and Fever in Neutropenic Patients with Multiple Myeloma Undergoing Autologous Hematopoietic Stem Cell Transplantation. Biology of Blood and Marrow Transplantation. 2015;21(10):1808-14.
37. Simondsen KA, Reed MP, Mably MS, Zhang Y, Longo WL. Retrospective analysis of fluoroquinolone prophylaxis in patients undergoing allogeneic hematopoietic stem cell transplantation. J Oncol Pharm Pract. 2013;19(4):291-7.
38. Zavrelova A, Paterova P, Gabalec F, Zak P, Radocha J. Ciprofloxacin prophylaxis during autologous stem cell transplantation for multiple myeloma in patients with a high rate of fluoroquinolone-resistant gram-negative bacteria colonization. Biomedical Papers-Olomouc. 2019;163(2):161-5.
39. Bartella AK, Kamal M, Teichmann J, Kloss-Brandstatter A, Steiner T, Holzle F, et al. Prospective comparison of perioperative antibiotic management protocols in oncological head and neck surgery. Journal of Cranio-Maxillofacial Surgery. 2017;45(7):1078-82.
40. Averbuch D, Tridello G, Hoek J, Mikulska M, Akan H, San Segundo LY, et al. Antimicrobial Resistance in Gram-Negative Rods Causing Bacteremia in Hematopoietic Stem Cell Transplant Recipients: Intercontinental Prospective Study of the Infectious Diseases Working Party of the European Bone Marrow Transplantation Group. Clinical Infectious Diseases. 2017;65(11):1819-28.
41. Verlinden A, Jansens H, Goossens H, van de Velde AL, Schroyens WA, Berneman ZN, et al. Clinical and microbiological impact of discontinuation of fluoroquinolone prophylaxis in patients with prolonged profound neutropenia. European Journal of Haematology. 2014;93(4):302-8.
42. Sohn BS, Yoon DH, Kim S, Lee K, Kang EH, Park JS, et al. The role of prophylactic antimicrobials during autologous stem cell transplantation: a single-center experience. European Journal of Clinical Microbiology & Infectious Diseases. 2012;31(7):1653-61.
43. Macesic N, Morrissey CO, Cheng AC, Spencer A, Peleg AY. Changing microbial epidemiology in hematopoietic stem cell transplant recipients: increasing resistance over a 9-year period. Transplant Infectious Disease. 2014;16(6):887-96.
44. Rubinstein E, Findler G, Amit P, Shaked I. Perioperative prophylactic cephazolin in spinal surgery. A double-blind placebo-controlled trial. Journal of bone and joint surgery British volume. 1994;76(1):99-102.
45. Saito T, Yoshioka S, Iinuma Y, Takakura S, Fujihara N, Ichinohe T, et al. Effects on spectrum and susceptibility patterns of isolates causing bloodstream infection by restriction of fluoroquinolone prophylaxis in a hematology-oncology unit. European Journal of Clinical Microbiology & Infectious Diseases. 2008;27(3):209-16.
46. Schroeder M, Schadeck-Gressel C, Selbach J, Westerhausen M. Antibiotic prophylaxis with gyrase inhibitors during cytostatically induced granulocytopenias in patients with solid tumors: a double-blind prospective randomized study. Onkologie. 1992;15(6):476-9.
47. Guiot HF, van der Meer JW, van den Broek PJ, Willemze R, van Furth R. Prevention of viridans-group streptococcal septicemia in oncohematologic patients: a controlled comparative study on the effect of penicillin G and cotrimoxazole. Ann Hematol. 1992;64(6):260-5.
48. Trecarichi EM, Giuliano G, Cattaneo C, Ballanti S, Criscuolo M, Candoni A, et al. Bloodstream infections caused by Escherichia coli in onco-haematological patients: Risk factors and mortality in an Italian prospective survey. Plos One. 2019;14(10).

49. Munoz L, Martino R, Subira M, Brunet S, Sureda A, Sierra J. Intensified prophylaxis of febrile neutropenia with ofloxacin plus rifampin during severe short-duration neutropenia in patients with lymphoma. Leukemia & Lymphoma. 1999;34(5-6):585-9.
50. Yang CH, Chew KY, Solomkin JS, Lin PY, Chiang YC, Kuo YR. Surgical Site Infections Among High-Risk Patients in Clean-Contaminated Head and Neck Reconstructive Surgery Concordance With Preoperative Oral Flora. Annals of Plastic Surgery. 2013;71:S55-S60.
51. Martino R, Subira M, Altes A, Lopez R, Sureda A, Domingo-Albos A, et al. Effect of discontinuing prophylaxis with norfloxacin in patients with hematologic malignancies and severe neutropenia - A matched case-control study of the effect on infectious morbidity. Acta Haematologica. 1998;99(4):206-11.
52. Wilson SE, Turpin RS, Kumar RN, Itani KM, Jensen EH, Pellissier JM, et al. Comparative costs of ertapenem and cefotetan as prophylaxis for elective colorectal surgery. Surg Infect (Larchmt). 2008;9(3):349-56.
53. Gentilotti E, De Nardo P, Nguhuni B, Piscini A, Damian C, Vairo F, et al. Implementing a combined infection prevention and control with antimicrobial stewardship joint program to prevent caesarean section surgical site infections and antimicrobial resistance: a Tanzanian tertiary hospital experience. Antimicrobial Resistance and Infection Control. 2020;9(1).
54. Lee DG, Choi SM, Choi JH, Yoo JH, Park YH, Kim YJ, et al. Selective bowel decontamination for the prevention of infection in acute myelogenous leukemia: a prospective randomized trial. The Korean journal of internal medicine. 2002;17(1):38-44.
55. Garnica M, Nouér SA, Pellegrino FL, Moreira BM, Maiolino A, Nucci M. Ciprofloxacin prophylaxis in high risk neutropenic patients: effects on outcomes, antimicrobial therapy and resistance. BMC Infect Dis. 2013;13:356.
56. D'Antonio D, Piccolomini R, Iacone A, Fioritoni G, Parruti G, Betti S, et al. Comparison of ciprofloxacin, ofloxacin and pefloxacin for the prevention of the bacterial infection in neutropenic patients with haematological malignancies. Journal of antimicrobial chemotherapy. 1994;33(4):837-44.
57. Nucci M, Pulcheri W, Spector N, Bueno A, Silveira S, Marangoni D, et al. Quinolone prophylaxis in neutropenic patients - efficacy versus resistance. Oncol Rep. 1994;1(6):1101-5.
58. Carena AA, Jorge L, Bonvehi P, Temporiti E, Zarate MS, Herrera F. Levofloxacin prophylaxis in neutropenic patients. Medicina-Buenos Aires. 2016;76(5):295-303.
59. Delarive P, Baumgartner JD, Glauser MP, Cometta A. Evaluation of antibiotic prophylaxis in neutropenic patients with haematological malignancies. Schweizerische Medizinische Wochenschrift. 2000;130(48):1837-44.
60. Slavin MA, Grigg AP, Schwarer AP, Szer J, Spencer A, Sainani A, et al. A randomized comparison of empiric or pre-emptive antibiotic therapy after hematopoietic stem cell transplantation. Bone Marrow Transplantation. 2007;40(2):157-63.
61. Mahida N, Boswell T. Fluoroquinolone prophylaxis in haematopoietic bone marrow transplantation: a driver for antimicrobial resistance. Journal of Hospital Infection. 2018;98(3):241-2.
62. Ugarte-Torres A, Villasis-Keever A, Hernandez-Bribiesca ME, Crespo-Solis E, Ruiz-Palacios GM, Sifuentes-Osornio J, et al. Fluoroquinolone prophylaxis utility during chemoradiation induced severe neutropenia in patients with acute leukemia, with fluoroquinolone resistance high prevalence, in a reference hospital in Mexico City. Revista De Investigacion Clinica-Clinical and Translational Investigation. 2006;58(6):547-54.
63. Sojo JF, Massana MB, Morgades M, Polo SV, Quesada MD, Santasusana JMR. Comparative study on the usefulness of antibacterial prophylaxis with levofloxacin in patients submitted to hematopoietic stem cell transplantation. Medicina Clinica. 2016;146(1):16-9.
64. Qin TJ, Mi YC, Feng SZ, Li DP, Wei JL, Yang DL, et al. [Clinical study on fluoroquinolone prophylaxis in neutropenia patients with acute leukemia]. Zhonghua Yi Xue Za Zhi. 2007;87(20):1389-93.

65. Sinkó J, Cser V, Konkoly Thege M, Masszi T. [Gram-negative bacteremia in neutropenic patients with hematologic disorders. Experiences with prophylactic use of fluoroquinolones]. Orv Hetil. 2011;152(27):1063-7.
66. Amelot A, Riche M, Latreille S, Degos V, Carpentier A, Mathon B, et al. Antimicrobial prophylaxis in noninstrumented spine surgery: a prospective study to determine efficacy and drawbacks. J Neurosurg Spine. 2021:1-10.
67. Higaki E, Abe T, Fujieda H, Hosoi T, Nagao T, Komori K, et al. Significance of Antimicrobial Prophylaxis for the Prevention of Early-Onset Pneumonia After Radical Esophageal Cancer Resection: A Retrospective Analysis of 356 Patients Undergoing Thoracoscopic Esophagectomy. Annals of Surgical Oncology. 2022;29(2):1374-87.
68. Caro J, Moshier E, Tremblay D, Coltoff A, Lancman G, Rana M, et al. Impact of Primary Antibacterial Prophylaxis on Neutropenic Fever, Infections, and Antimicrobial Resistance in Newly Diagnosed AML Patients. Blood. 2019;134:3849-.
69. Longtin Y, Gervais P, Birnie DH, Wang J, Alings M, Philippon F, et al. Impact of Choice of Prophylaxis on the Microbiology of Cardiac Implantable Electronic Device Infections: Insights From the Prevention of Arrhythmia Device Infection Trial (PADIT). Open Forum Infect Dis. 2021;8(11):ofab513.
70. Dubinsky-Pertzov B, Temkin E, Harbarth S, Fankhauser-Rodriguez C, Carevic B, Radovanovic I, et al. Carriage of Extended-spectrum Beta-lactamase-producing Enterobacteriaceae and the Risk of Surgical Site Infection After Colorectal Surgery: A Prospective Cohort Study. Clin Infect Dis. 2019;68(10):1699-704.
71. Satlin MJ, Chen L, Douglass C, Hovan M, Davidson E, Soave R, et al. Colonization With Fluoroquinolone-Resistant Enterobacterales Decreases the Effectiveness of Fluoroquinolone Prophylaxis in Hematopoietic Cell Transplant Recipients. Clin Infect Dis. 2021;73(7):1257-65.
72. Nutman A, Temkin E, Harbarth S, Carevic B, Ris F, Fankhauser-Rodriguez C, et al. Personalized Ertapenem Prophylaxis for Carriers of Extended-spectrum β-Lactamase-producing Enterobacteriaceae Undergoing Colorectal Surgery. Clin Infect Dis. 2020;70(9):1891-7.
73. Clerici D, Galli L, Greco R, Lugli AP, Erbella F, Ripa M, et al. Levofloxacin prophylaxis vs no prophylaxis in patients with neutropenia within an endemic country for carbapenem-resistant GNB. Blood Advances. 2023;7(9):1621-34.
74. Guare EG, Hale CM, Sivik J, Lehman E, Inoue Y, Rakszawski K, et al. The addition of doxycycline to fluoroquinolones for bacterial prophylaxis in autologous stem cell transplantation for multiple myeloma. Transplant Infectious Disease. 2024;26(2).
75. Urbino I, Frairia C, Busca A, Corcione S, D'Ardia S, Dellacasa CM, et al. Levofloxacin Prophylaxis Versus no Prophylaxis in Acute Myeloid Leukemia During Post-Induction Aplasia: a Single Center Study. Mediterr J Hematol Infect Dis. 2023;15(1):e2023022.
76. Guimaraes T, Borges IC, Spadao FD, Mariano L, Nascimento MD, Higashino H, et al. Impact of Discontinuing Levofloxacin Prophylaxis on Bloodstream Infections in Neutropenic Hematopoietic Stem Cell Transplantation Patients. Antibiotics-Basel. 2022;11(9).
77. Caro J, Madero-Marroquin R, Zubizarreta N, Moshier E, Tremblay D, Coltoff A, et al. Impact of Fluoroquinolone Prophylaxis on Neutropenic Fever, Infections, and Antimicrobial Resistance in Newly Diagnosed AML Patients. Clin Lymphoma Myeloma Leuk. 2022;22(12):903-11.
78. Mestrallet P, Yanni A, Roman A, Rodriguez A, Bouland C, Javadian R, et al. Antibiotic use in patients undergoing complex clean-contaminated head and neck surgery: A prospective study. Journal of International Society of Preventive and Community Dentistry. 2023;13(4):349-55.
79. Khawcharoenporn T, Kanoktipakorn P. Effectiveness of appropriate antibiotic prophylaxis for transurethral resection of the prostate in the era of antibiotic resistance. Infect Control Hosp Epidemiol. 2022;43(11):1693-7.
80. Kuo FC, Chang YH, Huang TW, Chen DW, Tan TL, Lee MS. Post-operative prophylactic antibiotics in aseptic revision hip and knee arthroplasty: a propensity score matching analysis. Sci Rep. 2022;12(1):18319.

81. Akhmedov M, Klyasova G, Kuzmina L, Fedorova A, Drokov M, Parovichnikova E. Impact of fluoroquinolone administration and gut mucosal colonization on the risk of pre-engraftment bloodstream infections after allogeneic hematopoietic cell transplantation. Leuk Lymphoma. 2023;64(6):1102-11.
82. Newman TH, Stroman L, Hadjipavlou M, Haque A, Rusere J, Chan K, et al. EXIT from TRansrectal prostate biopsies (TREXIT): sepsis rates of transrectal biopsy with rectal swab culture guided antimicrobials versus freehand transperineal biopsy. Prostate Cancer Prostatic Dis. 2022;25(2):283-7.
83. Sewick A, Makani A, Wu C, O'Donnell J, Baldwin KD, Lee GC. Does dual antibiotic prophylaxis better prevent surgical site infections in total joint arthroplasty? Clin Orthop Relat Res. 2012;470(10):2702-7.
84. Bains SS, Dubin JA, Hameed D, Chen Z, Moore MC, Shrestha A, et al. Addition of vancomycin to cefazolin is often unnecessary for preoperative antibiotic prophylaxis during total joint arthroplasties. Arthroplasty. 2024;6(1):20.
85. Bartella AK, Lemmen S, Burnic A, Kloss-Brandstatter A, Kamal M, Breisach T, et al. Influence of a strictly perioperative antibiotic prophylaxis vs a prolonged postoperative prophylaxis on surgical site infections in maxillofacial surgery. Infection. 2018;46(2):225-30.
86. Lopez WY, Rider SM, Nwosu K, Kazarian ER, Blucher JA, Schoenfeld EM, et al. The Impact of Vancomycin and Cefazolin as Standard Preoperative Antibiotic Prophylaxis on Surgical Site Infections Following Instrumented Spinal Fusion. Spine. 2019;44(6):E366-E71.
87. Finkelstein R, Rabino G, Mashiah T, Bar-El Y, Adler Z, Kertzman V, et al. Vancomycin versus cefazolin prophylaxis for cardiac surgery in the setting of a high prevalence of methicillin-resistant staphylococcal infections. J Thorac Cardiovasc Surg. 2002;123(2):326-32.
88. Ishibashi K, Kuwabara K, Ishiguro T, Ohsawa T, Okada N, Miyazaki T, et al. Short-term intravenous antimicrobial prophylaxis in combination with preoperative oral antibiotics on surgical site infection and methicillin-resistant Staphylococcus aureus infection in elective colon cancer surgery: results of a prospective randomized trial. Surg Today. 2009;39(12):1032-9.
89. Ishida H, Yokoyama M, Nakada H, Inokuma S, Hashimoto D. Impact of oral antimicrobial prophylaxis on surgical site infection and methicillin-resistant Staphylococcus aureus infection after elective colorectal surgery. Results of a prospective randomized trial. Surg Today. 2001;31(11):979-83.
90. Kanayama M, Hashimoto T, Shigenobu K, Oha F, Togawa D. Effective prevention of surgical site infection using a Centers for Disease Control and Prevention guideline-based antimicrobial prophylaxis in lumbar spine surgery. J Neurosurg Spine. 2007;6(4):327-9.
91. Kusachi S, Sumiyama Y, Nagao J, Arima Y, Yoshida Y, Tanaka H, et al. Prophylactic antibiotics given within 24 hours of surgery, compared with antibiotics given for 72 hours perioperatively, increased the rate of methicillin-resistant Staphylococcus aureus isolated from surgical site infections. J Infect Chemother. 2008;14(1):44-50.
92. Liu C, Kakis A, Nichols A, Ries MD, Vail TP, Bozic KJ. Targeted Use of Vancomycin as Perioperative Prophylaxis Reduces Periprosthetic Joint Infection in Revision TKA. Clinical Orthopaedics and Related Research. 2014;472(1):227-31.
93. Mathur P, Trikha V, Farooque K, Sharma V, Jain N, Bhardwaj N, et al. Implementation of a short course of prophylactic antibiotic treatment for prevention of postoperative infections in clean orthopaedic surgeries. Indian Journal of Medical Research. 2013;137:111-6.
94. Marigi IM, Yu K, Nieboer M, Marigi EM, Sperling JW, Sanchez-Sotelo J, et al. After Primary Shoulder Arthroplasty Appropriate Vancomycin Antibiotic Prophylaxis Does Not Lead to Increased Infectious Complications When Compared to Cefazolin. J Shoulder Elbow Surg. 2024.
95. Peel TN, Astbury S, Cheng AC, Paterson DL, Buising KL, Spelman T, et al. Trial of Vancomycin and Cefazolin as Surgical Prophylaxis in Arthroplasty. N Engl J Med. 2023;389(16):1488-98.
96. Stallard S, Savioli F, McConnachie A, Norrie J, Dudman K, Morrow ES, et al. Antibiotic prophylaxis in breast cancer surgery (PAUS trial): randomised clinical double-blind parallel-group multicentre superiority trial. British Journal of Surgery. 2022;109(12):1224-31.

97. Nguyen AV, Coggins WS, Jain RR, Branch DW, Allison RZ, Maynard K, et al. Cefazolin versus vancomycin for neurosurgical operative prophylaxis - A single institution retrospective cohort study. Clin Neurol Neurosurg. 2019;182:152-7.
98. Cao Y, Pu K, Li G, Yan X, Ma Y, Xue K, et al. The Role of Antibiotic Prophylaxis in Clean Neurosurgery. World Neurosurg. 2017;100:305-10.
99. Manecksha RP, Nason GJ, Cullen IM, Fennell JP, McEvoy E, McDermott T, et al. Prospective study of antibiotic prophylaxis for prostate biopsy involving >1100 men. ScientificWorldJournal. 2012;2012:650858.
100. Patrick S, James C, Ali A, Lawson S, Mary E, Modak A. Vascular surgical antibiotic prophylaxis study (VSAPS). Vasc Endovascular Surg. 2010;44(7):521-8.
101. Fahmy AM, Kotb A, Youssif TA, Abdeldiam H, Algebaly O, Elabbady A. Fosfomycin antimicrobial prophylaxis for transrectal ultrasound-guided biopsy of the prostate: A prospective randomised study. Arab J Urol. 2016;14(3):228-33.
102. Pace G, Carmignani L, Marenghi C, Mombelli G, Bozzini G. Cephalosporins periprostatic injection: are really effective on infections following prostate biopsy? Int Urol Nephrol. 2012;44(4):1065-70.
103. Ongun S, Aslan G, Avkan-Oguz V. The effectiveness of single-dose fosfomycin as antimicrobial prophylaxis for patients undergoing transrectal ultrasound-guided biopsy of the prostate. Urol Int. 2012;89(4):439-44.
104. Haga N, Ishida H, Ishiguro T, Kumamoto K, Ishibashi K, Tsuji Y, et al. A prospective randomized study to assess the optimal duration of intravenous antimicrobial prophylaxis in elective gastric cancer surgery. Int Surg. 2012;97(2):169-76.
105. Ho HSS, Ng LG, Tan YH, Yeo M, Cheng CWS. Intramuscular Gentamicin Improves the Efficacy of Ciprofloxacin as an Antibiotic Prophylaxis for Transrectal Prostate Biopsy. Ann Acad Med Singap. 2009;38(3):212-6.
106. Lista F, Redondo C, Meilán E, García-Tello A, de Fata FR, Angulo JC. Efficacy and safety of Fosfomycin-trometamol in the prophylaxis for transrectal prostate biopsy. Prospective randomized comparison with ciprofloxacin. Actas Urol Esp. 2014;38(6):391-6.
107. Mohri Y, Tonouchi H, Kobayashi M, Nakai K, Kusunoki M, Mie Surgical Infection Research G. Randomized clinical trial of single- versus multiple-dose antimicrobial prophylaxis in gastric cancer surgery. Br J Surg. 2007;94(6):683-8.
108. De Pastena M, Paiella S, Azzini AM, Zaffagnini A, Scarlini L, Montagnini G, et al. Antibiotic Prophylaxis with Piperacillin-Tazobactam Reduces Post-Operative Infectious Complication after Pancreatic Surgery: An Interventional, Non-Randomized Study. Surgical Infections. 2021;22(5):536-42.
109. Tang B, Liu X, Xing F, Wang C, Jia C, Peng S, et al. Single Dose Based Ertapenem Prophylaxis Reduces Surgical Site Infection after Selective Hepatectomy of Hepatocellular Carcinoma: A Propensity Score Matching Study. Biomed Res Int. 2018;2018:2520191.
